# High-resolution structural and functional deep brain imaging using adaptive optics three-photon microscopy

**DOI:** 10.1038/s41592-021-01257-6

**Published:** 2021-09-30

**Authors:** Lina Streich, Juan Carlos Boffi, Ling Wang, Khaleel Alhalaseh, Matteo Barbieri, Ronja Rehm, Senthilkumar Deivasigamani, Cornelius T. Gross, Amit Agarwal, Robert Prevedel

**Affiliations:** 1 Cell Biology and Biophysics Unit, European Molecular Biology Laboratory (EMBL), Heidelberg, Germany; 2 Collaboration for joint PhD degree between EMBL and Heidelberg University, Faculty of Biosciences, Heidelberg University, Heidelberg, Germany; 3 The Chica and Heinz Schaller Research Group, Institute for Anatomy and Cell Biology, Heidelberg University, Heidelberg, Germany; 4 Epigenetics and Neurobiology Unit, European Molecular Biology Laboratory, Monterotondo, Italy; 5 Interdisciplinary Center for Neurosciences, Heidelberg University, Heidelberg, Germany; 6 Developmental Biology Unit, European Molecular Biology Laboratory, Heidelberg, Germany; 7 Molecular Medicine Partnership Unit (MMPU), European Molecular Biology Laboratory, Heidelberg, Germany

**Keywords:** Glial biology, Multiphoton microscopy, Fluorescence imaging, Ca2+ imaging

## Abstract

Multiphoton microscopy has become a powerful tool with which to visualize the morphology and function of neural cells and circuits in the intact mammalian brain. However, tissue scattering, optical aberrations and motion artifacts degrade the imaging performance at depth. Here we describe a minimally invasive intravital imaging methodology based on three-photon excitation, indirect adaptive optics (AO) and active electrocardiogram gating to advance deep-tissue imaging. Our modal-based, sensorless AO approach is robust to low signal-to-noise ratios as commonly encountered in deep scattering tissues such as the mouse brain, and permits AO correction over large axial fields of view. We demonstrate near-diffraction-limited imaging of deep cortical spines and (sub)cortical dendrites up to a depth of 1.4 mm (the edge of the mouse CA1 hippocampus). In addition, we show applications to deep-layer calcium imaging of astrocytes, including fibrous astrocytes that reside in the highly scattering corpus callosum.

## Abstract (web summary)

Three-photon microscopy in combination with adaptive optics-based aberration correction and ECG-triggered gating allows high-resolution imaging of neurons and astrocytes up to a depth of 1.4 mm in the mouse brain.

## Main

In scattering tissues such as the mammalian brain, two-photon excitation microscopy (2PM) is the gold standard for recording cellular structure and function in noninvasive and physiologically relevant conditions in vivo^1,2^. However, the maximum penetration depth of two-photon microscopes is fundamentally limited by the onset of out-of-focus fluorescence near the surface with increasing excitation power, which for the mammalian brain prevents imaging beyond roughly 1 mm (ref. ^3^). Imaging approaches based on three-photon excitation microscopy (3PM) have shown potential for deep imaging beyond 1 mm with cellular resolution, owing to a substantially increased signal-to-background ratio (SBR) at depth and longer wavelength excitation^4–7^.

As in 2PM, however, with increasing imaging depths optical aberrations due to tissue heterogeneities and refractive index mismatches, and subtle motion artifacts due to the animal’s heartbeat, degrade image resolution and overall performance, and result in a loss of subcellular details. This has so far prohibited the resolution of fine neuronal processes, synapses and subcellular Ca^2+^ transients in deep cortical and subcortical areas of the mouse brain in vivo without using highly invasive methods such as gradient index lens implantation^8^ or cortical aspiration^9^. While optical aberrations can be measured and compensated by using adaptive optics (AO) methods^10,11^, previous implementations were predominantly based on 2PM and thus were limited in their effective imaging depth to at most 800 µm in the mouse brain^12–15^. Alternative approaches based on wavefront shaping have the potential to image even further into highly scattering media, but limited fields of view (FOV) of only tens of micrometers and/or fast decorrelation times have prohibited their useful application in realistic intravital conditions^16–18^.

A further challenge in in vivo deep brain imaging is that at large tissue depths, heart pulsation leads to intraframe motion artifacts that prevent frame averaging to enhance the signal-to-noise ratio (SNR), a standard technique that is essential to reliably resolve small structures such as dendrites and individual spines.

To address the above shortcomings, we developed a minimally invasive intravital imaging methodology based on 3PM, indirect AO correction and active electrocardiogram (ECG) gating to achieve aberration correction and near-diffraction-limited resolution up to a depth of over 1.4 mm in the mouse brain. This enabled us to resolve individual synapses down to roughly 900 µm in the cortex, and fine dendritic processes in the hippocampus at a depth of more than 1.4 mm. Furthermore, our noninvasive approach achieved in vivo functional characterization of fibrous astrocytes in the white matter and resolved Ca^2+^ transients in individual microdomains.

## Results

### 3PM with ECG gating

**Fig. 1:**
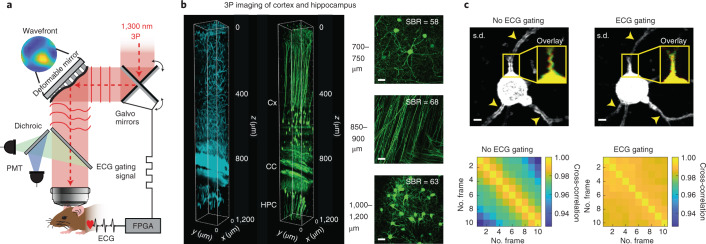
Schematic principle of ECG-gated AO 3PM. **a**, Illustration of the motion-corrected AO 3P microscope. Aberration correction is performed via a modal-based indirect wavefront sensing approach. Intravital motion artifacts are reduced with a real-time ECG-gated image acquisition scheme that synchronizes the Galvo scanners to the cardiac cycle of the mouse. **b**, 3PM at 1,300 nm excitation wavelength in EGFP–Thy1(M) mouse visual cortex and hippocampus. Left, 3D reconstruction of a 3P image stack of third-harmonic signal (cyan) and GFP-labeled neurons (green). Right, maximum intensity projection images at various depths in the cortex (Cx; top), corpus callosum (CC; middle) and CA1 region of the hippocampus (HPC; bottom). SBRs at different depths are displayed in the respective images. Scale bar, 20 μm. **c**, Comparison of intraframe motion artifacts for ECG-gated and nongated image acquisition at 701 μm depth. Standard deviation (s.d.) projection images of consecutively acquired frames with (top, right) and without (top, left) ECG gating. Arrows indicate high frame-to-frame variability resulting in artifacts without ECG gating. Yellow box shows overlay of two consecutively acquired frames (red and green). Bottom, pairwise two-dimensional cross-correlation between individual frames without (bottom, left) and with (bottom, right) ECG synchronization. Representative datasets obtained from *n* = 4 mice. Source data

**Extended Data Fig. 1:**
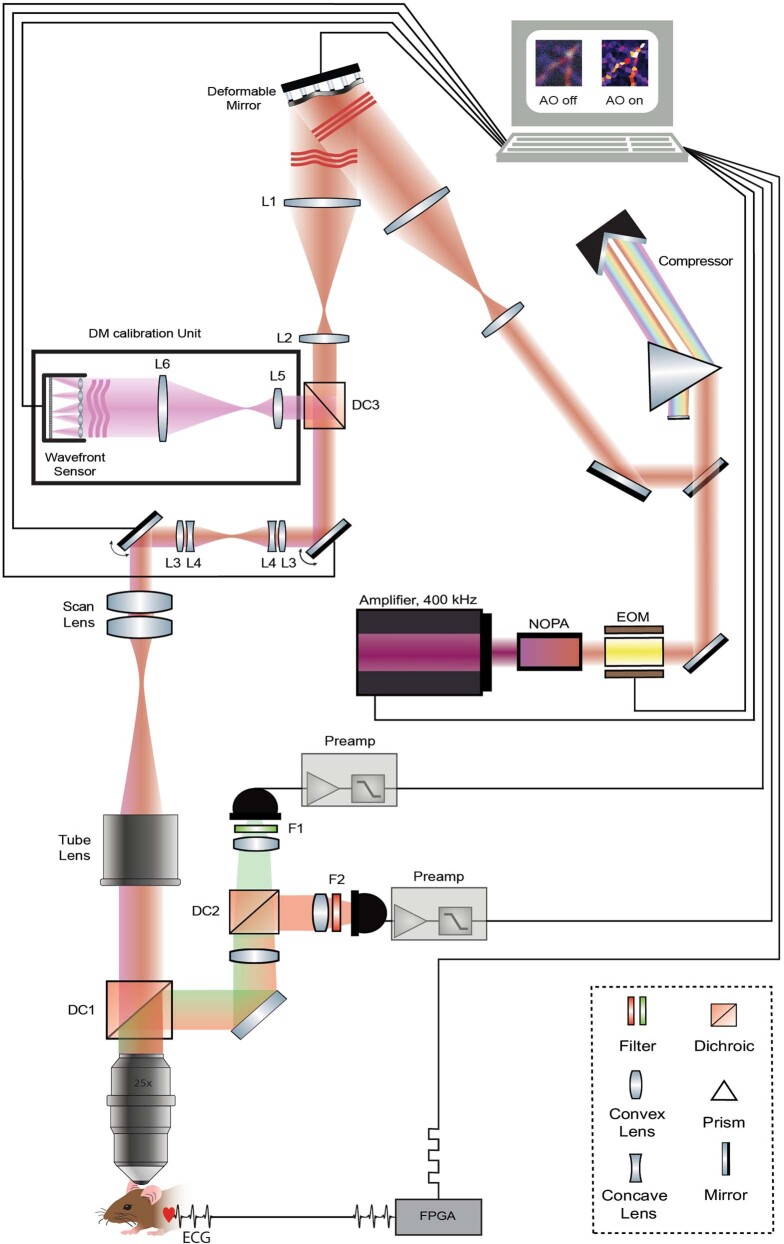
Schematic experimental set-up. Femto-second laser pulses at 1300 nm are generated by a NOPA system pumped by a fiber-based amplified laser. Laser pulse duration is maintained at the sample plane with a custom-build single-prism pulse compressor. The deformable mirror, X and Y galvo mirrors, and the wavefront sensor are conjugated to the objective pupil plane with 4-f relay lens pairs. Fluorescence signals are collected by large-aperture optics and detected by large-area PMTs. Physiological parameters of the mouse (ECG, breath rate, etc.) are processed in real-time by an FPGA which in turn gates the image acquisition of the microscope.

Our intravital imaging method is based on a homebuilt 3P laser scanning microscope optimized for 1,300 nm excitation and the use of broad-bandwidth, low repetition-rate lasers (Fig. 1a and Extended Data Fig. 1). We optimized the 3P fluorescence signal by ensuring short-duration laser pulses (<50 fs) and efficient power delivery to the focal plane within the sample. Optimization of the in vivo fluorescence signal in 3PM is especially important to avoid laser-induced tissue heating or long-term tissue damage^7,19–21^.

**Extended Data Fig. 2:**
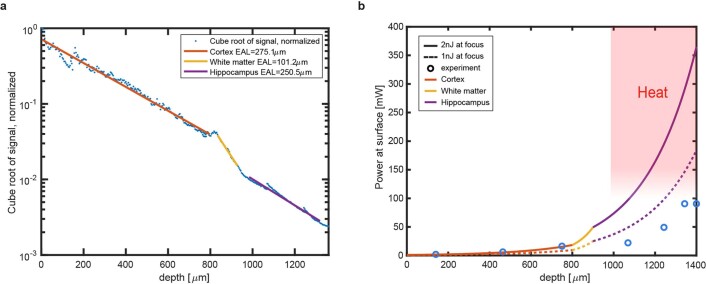
Power attenuation and estimated focal energies at depth with 1300 nm excitation of brain tissue including cortex, corpus callosum (white matter) and hippocampus. **a**, THG signal attenuation vs depth in the mouse brain with fits displaying attenuation length (EAL) in the cortex (red, EAL = 275μm), white matter (yellow, EAL = 101μm) and hippocampus (purple, EAL = 250μm). The brightest 0.5% of pixels of each THG image slice were averaged. The cube root of the resulting average was scaled by the surface power, and the entire curve was normalized by the maximum. **b**, Estimated theoretical focal energy (2nJ straight line and 1nJ dotted line) for a given surface power P_0_ based on attenuation length determined in (a) (P(z) = P_0_*exp(-z/EAL). Experimental power values used for Extended Data Fig. 3 are plotted as blue dots. Heating threshold taken from Ref. ^21^. Source data

We first validated the deep-tissue capability of our 3P microscope by imaging neuronal structure and third-harmonic generation contrast along an entire cortical column and into the hippocampus up to 1.2 mm depth in a Thy1EGFP-M (EGFP, enhanced green fluorescent protein) mouse through a chronic glass window (Fig. 1b and Supplementary Video 1). We observed high SBRs, even at deep, subcortical layers below the white matter, while ensuring physiologically low (0.5–22 mW) average laser power and focal energies (<2 nJ) below previously established damage and heating thresholds^7,21^ (Extended Data Fig. 2).

Despite the high SBR as well as numerical aperture (NA) and thus spatial resolution of our 3P system, however, fine neuronal processes such as dendritic spines are sometimes only reliably resolved down to roughly 500 µm, and completely disappear below the highly scattering corpus callosum. One possible reason for this is residual physiological brain motion resulting from cardiac activities, which results in intraframe imaging artifacts that are difficult to correct during postprocessing (Supplementary Video 2). These distortions typically increase with depth and distance from the stabilizing cranial window and headbar, and can lead to image blurring when using frame averaging, as typically done to increase image SBR. Therefore, they present an inherent challenge for in vivo high-resolution intravital microscopy, especially at greater depth^22^.

To address this, we implemented a prospective image-gated acquisition scheme based on a field-programmable gate array (FPGA), which enables active synchronization of the scanners to the cardiac cycle in real time so that active scanning is paused during peaks of ECG recording. We found that this straightforward ECG-gating approach substantially reduced intraframe motion artifact down to the hippocampus (>1 mm depth), and was most useful at large tissue depths and for visualizing fine structures such as individual spines. Furthermore, temporal sequences of frames acquired with ECG gating were more correlated to one another than were non-gated frames, indicating higher temporal image stability (Fig. 1c, Supplementary Fig. 1 and Supplementary Video 2).

Compared to previous work that used ECG-triggered full frame acquisition^23,24^, our approach is independent of the chosen acquisition parameters, does not require additional postprocessing and can readily be applied to large-FOV scanning. While we note that our ECG gating effectively reduces the duty cycle of the image acquisition by 40–60% and works only with galvanometer-based scanners, which poses limitations for recordings of fast dynamics, it is also a considerably simpler and sample-independent solution compared to more elaborate active motion correction techniques^25–27^.

### Modal-based sensorless AO for deep imaging

A major challenge in multiphoton microscopy is degradation of the excitation point spread function (PSF) due to optical aberrations. This is particularly problematic in 3PM, as the fluorescence depends nonlinearly on the focal intensity^4^. Furthermore, submicrometer structures such as dendrites and spines are smaller than the axial extent of the PSF, and thus become even more challenging to resolve. Optical aberrations further increase the axial extent of the PSF, which leads to overall low fluorescence signal from fine structures. While 3PM has been used for deep cortical and subcortical imaging^5,7,28^, these previous studies focused on cellular (functional) investigations that did not require subcellular details.

To restore near-diffraction-limited resolution in deep brain regions, we incorporated indirect AO to our intravital ECG-gated 3PM. Many indirect, ‘sensorless’ AO approaches have been developed for microscopy over the years, including so-called ‘modal’ methods^29,30^, where the aberrations are represented by a series of modes defined over the whole pupil, and various ‘pupil-segmented’ approaches^31,32^, in which the pupil is separated into a number of zones that are modulated separately. We chose indirect, modal-based AO^30^ as these approaches were predicted to be most robust in low-signal and high-noise conditions^33^ and are thus arguably best suited to push the depth limits in deep brain imaging, especially when combined with higher-order multiphoton microscopy^34^.

**Fig. 2:**
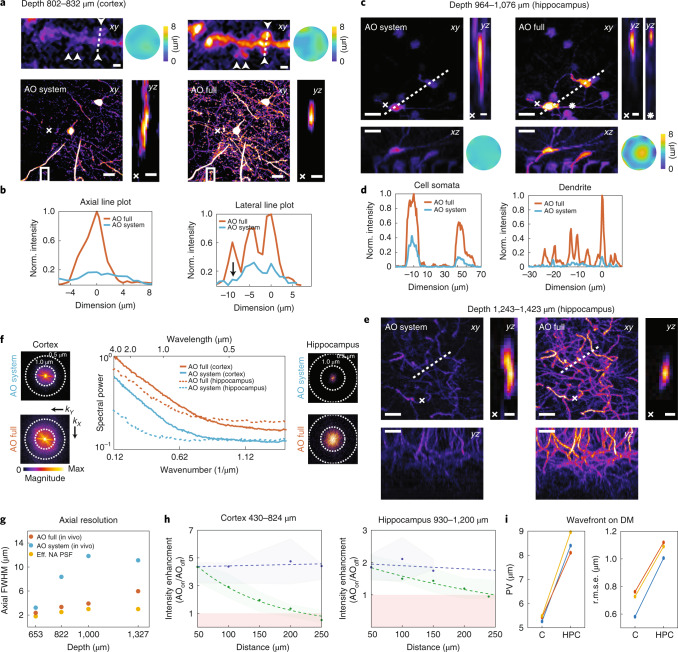
AO enables high-resolution 3P deep brain imaging. Indirect modal-based AO correction and ECG-gated 3PM at 1,300 nm excitation. **a**,**c**,**e**, Representative images showing dendrites and spines in (**a**) layer VI and (**c**,**e**) hippocampus of the in vivo mouse brain (EGFP–Thy1(M)) through a cranial window. Images were recorded with two different conditions: AO system (wavefront correction of system aberrations) and AO full (wavefront correction of system and brain tissue aberrations). Maximum intensity projection images with system and full aberration correction at layer VI in cortex (**a**) as well as hippocampus (**c**,**e**) (scale bar, 20 μm; on zoomed regions, 2 µm). White boxes in **a** indicate magnified views of spines. The white cross in **a**,**c**,**e** shows orthogonal view along dendrite. Respective wavefront maps for aberration correction are displayed in the top and bottom corners. Aberration measurement location for **e** was identical to **c**. **b**, Intensity profile along spines (right; white dotted line) and across dendrite (left; white cross), respectively, in cortical layer VI (**a**). Norm., normalized. **d**, Intensity profile along neuron somata (white dotted line in **c**) and across dendrite (white dotted line in **e**). **f**, Lateral resolution analysis of AO correction improvement. Spectral power map as a function of spatial frequency (wavenumber) at (left) layer VI of cortex (**a**) and (right) hippocampus (**e**). Middle, average radial profile of spectral power maps for maximum intensity images. **g**, Axial resolution analysis. Effective PSF of microscope inferred from beads in solution and compared to in vivo measurements performed on dendrites, with system and full AO at a depth of 653 μm (cortex), 822 μm (cortex), and 1,000 and 1,327 μm (hippocampus). For AO at 1,327 µm, the AO measurement was performed at 1,000 µm depth. The effective illumination NA was reduced at a greater depth to increase objective transmission. **h**, Spatial analysis of lateral (green) and axial (blue) intensity enhancement (AO on/AO off) with respect to distance from location of AO measurement in cortex (left) and hippocampus (right). Median values are shown as dots and top 75th and bottom 25th percentile as shadows. Dotted lines show exponential fits. The red box indicates the area where the intensity enhancement (AO on/AO off) <1. Same data as Extended Data Figs. 6 and 7 for Z35. **i**, Analysis of wavefront distortion (peak-to-valley (PV) and root-mean-square error (r.m.s.e.)) displayed on DM for aberration correction in cortex (C) and hippocampus (HPC) and pooled from Extended Data Fig. 8. Only datasets with <100 μm lateral off-set between cortex and hippocampus in the same animal were included in the analysis. Representative datasets obtained from several imaging sessions in *n* = 7 mice. Source data

**Extended Data Fig. 3:**
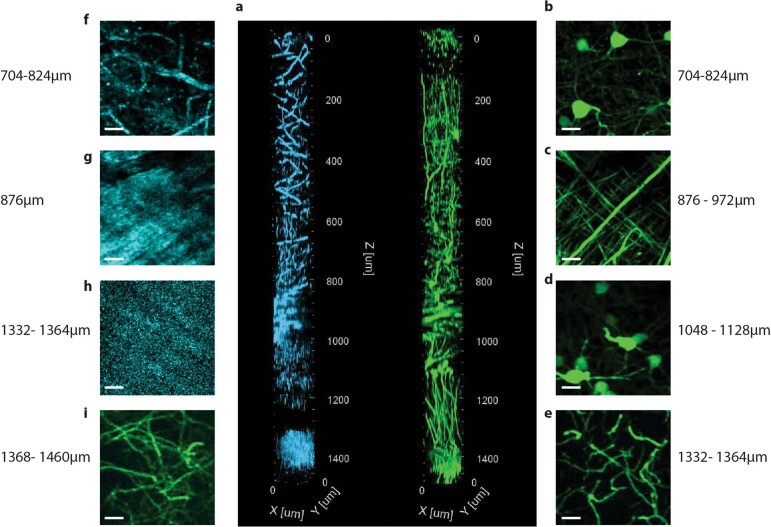
Three-photon adaptive optics microscopy at 1300 nm excitation of intact Thy1-GFP mouse brain down to the edge of the CA1 stratum lacunosum at ~1.45 mm depth. **a**, 3D reconstruction of three-photon image stack of THG signal (cyan) and GFP-labelled neurons (green). Image stack was acquired with AO correction based on AO measurements at three different depths (system 0 µm, cortex 600 µm, hippocampus 1000 µm), and utilized AO system correction for 0-500μm depth, AO Z35 (cortex) for 500-900μm depth, and AO Z35 (hippocampus) for 900-1450um depth. Lateral FOV 126×126 μm. **b-i**, Maximum intensity projection images of GFP (green) and THS (cyan) at various depths. **b,f**, Layer V/VI cortex where pyramidal cell bodies are imaged. **c,g**, At ~876-972μm depth the fibres of the corpus callosum (white matter) are visible. **d**, The CA1 pyramidal cell somata are visualized at ~1048-1128μm depth in the stratum pyramidale. **e,h,i**, At depth >1332μm the edge of the CA1 stratum lacunosum moleculare is reached, and the THS signal indicates denser tissue which suggests reaching the edge of the outer molecular layer or envelope of the hippocampal sulcus **(h)**. Depth values correspond to axial motor position. Taking the refractive index difference between the coverslip/immersion media and various brain tissue into account, the actual imaging depth is ~5-10% larger^4^. Representative result of (n=3) experiments.

**Extended Data Fig. 4:**
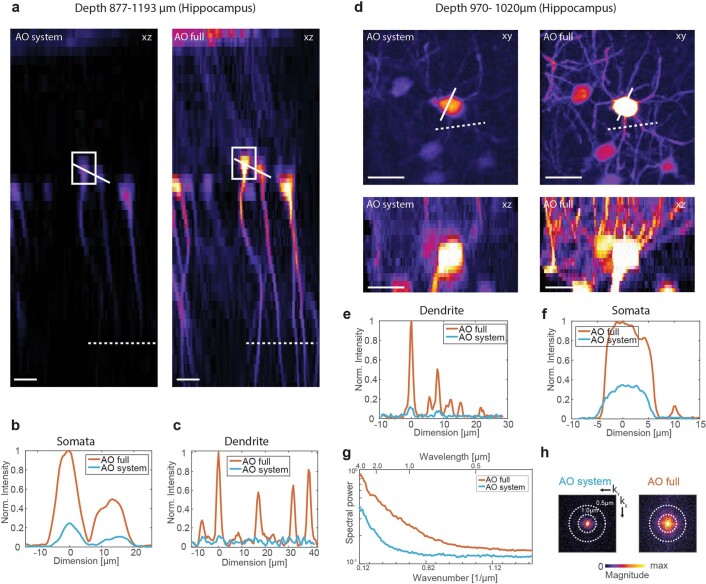
Image quality improvement for in-vivo imaging with aberration correction in Hippocampus. Images were recorded in Thy1-EGFP(M) mice with two different conditions. AO system: wavefront correction of system aberrations; AO full: wavefront correction of system and brain tissue aberrations including Z35 modes (excluding piston, tip and tilt) for correction. Additionally, cardiac gated image acquisition was performed for both AO system and AO full condition. **a**, Maximum intensity projection images of orthogonal view (xz) for the two different conditions at 877-1193μm depth. AO measurement was performed on somata indicated by white box. After aberration correction and intensity enhancement was achieved over the entire FOV, illustrated by intensity line plots across somata **(b)** and dendrites **(c)** indicated in (a) by straight and dotted white line, respectively. **d**, Maximum intensity projection images (top) and orthogonal view (bottom) for system and full AO correction at 970-1020μm depth. **(e)** and **(f)** display lateral intensity plot along dendrites and somata, respectively, indicated in (d) by white dotted and white straight line, respectively. **h**, Spectral power map as a function of spatial frequency (wavenumber) and **(g)** average radial profile of spectral power maps for maximum intensity images in (d). Scale bar 20μm. Representative result of (n=3) and (n=8) experiments in (a-b), respectively. Source data

We developed a continuous membrane deformable mirror- (DM-) and modal-based AO optimization scheme with automatic shift correction (Supplementary Fig. 2) and integrated it into the hardware control of our 3PM. First, we validated the AO performance by correcting artificial aberrations in ex vivo brain slices (Supplementary Fig. 3). Next, we applied our method to in vivo 3P imaging of neuronal structures throughout the cerebral cortex of Thy-1 EGFP-M mice (Fig. 2a,b and Supplementary Figs. 4–6 and Supplementary Videos 3 and 4). Moreover, with AO correction we were able to observe hippocampus CA1 neurons and dendrites down to the stratum lacunosum moleculare in the dorsal hippocampus at roughly 1.45 mm depth (Fig. 2c–e, Extended Data Figs. 3 and 4 and Supplementary Videos 5 and 6).

We observed substantial improvements in image quality and the spatial resolution due to AO correction. We directly measured up to fourfold improvement in effective axial resolution and about eightfold enhancements of fluorescence signals, as evidenced by axial intensity line plots and spectral power map analysis of lateral resolution (Fig. 2b,d,f,g and Supplementary Fig. 5). Here, higher spatial frequencies, *k*, were substantially restored by full AO correction, as can be seen by comparing the power in the spatial frequency distributions between full AO and systems AO correction. In addition, we note that the reported improvements in signal and resolution (AO full) are solely due to correction of tissue-induced aberration, since values are compared to the best possible performance of the microscope alone, including aberrations due to sample preparation (AO system).

With our 3P-AO scope, we were able to recover near-diffraction-limited performance at all imaging depths (Fig. 2g), and in particular to reliably resolve individual synapses down to roughly 900 µm in the cortex (Fig. 2a, Supplementary Fig. 4 and Supplementary Videos 3 and 4) and fine dendritic processes in the hippocampus at up to 1.4 mm depth (Fig. 2e, Supplementary Fig. 6, Extended Data Fig. 4 and Supplementary Video 6).

**Extended Data Fig. 5:**
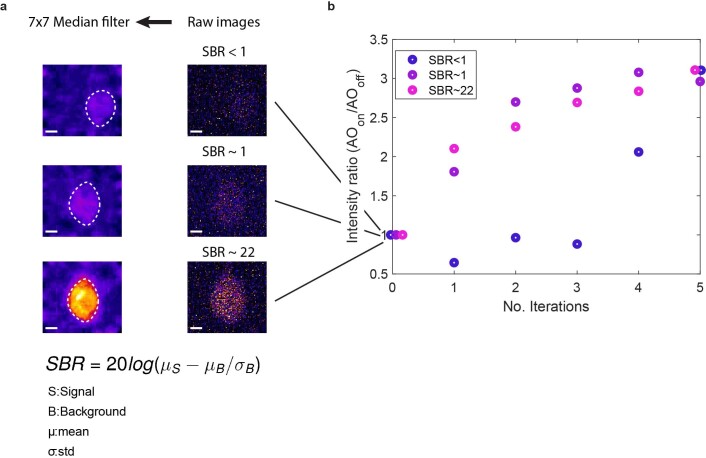
Characterization of initial SBR condition on AO measurement performance in-vivo at ~600μm in Thy1-GFP mouse at 1300 nm excitation. **a**, Initial image quality of neuron somata used for AO measurement under three different SBR conditions calculated on raw images (Right). For calculation of the image quality metric the raw images were (Left) median filtered (7x7pixel kernel) and thresholded so that only the somata signal (indicated by white dotted circle) was used for analysis. **b**, Intensity ratio (AO on/AO off) versus iteration number for the three different initial SBR conditions. Source data

We also characterized the influence of the starting SBR on the AO performance and found that our AO routine led to signal enhancement even at low starting SBR (Extended Data Fig. 5). This is a distinctive asset of our nonsegmented, modal AO technique in which the entire pupil is modulated at once, since such a strategy leads to larger AO-induced signal changes compared to segmented AO strategies^31,35,36^. Therefore, they are likely to be more robust at, and therefore better suited for, the low SBR conditions typically encountered in deep-tissue imaging, consistent with previous findings^33^.

### Spatial characterization of aberrations and AO performance

**Extended Data Fig. 6:**
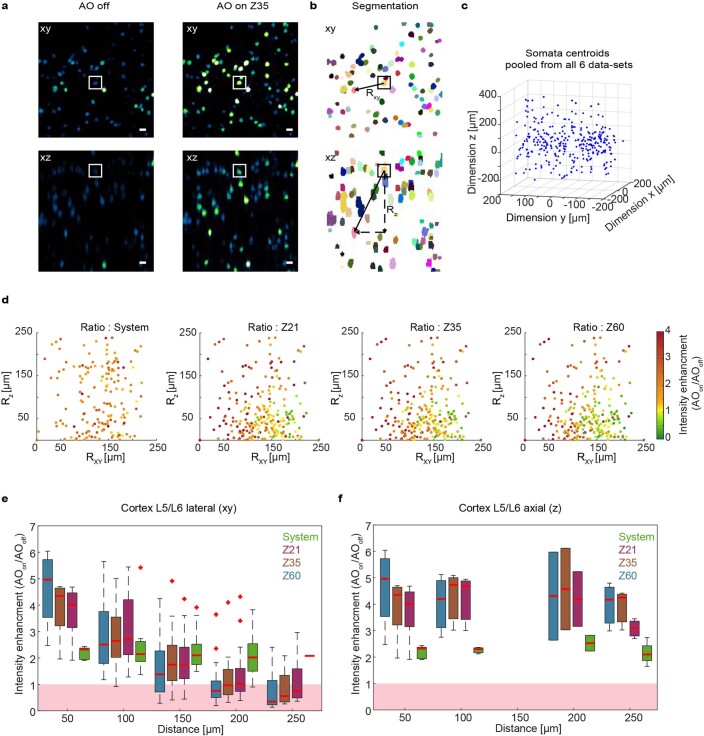
Spatial AO enhancement analysis for local aberration correction in-vivo in Thy1-GFP mouse cortex. Aberration measurement was performed for four different conditions referred to: system aberration correction (AO system) and full aberration correction inside cortex including Zernike modes up to order 21, (Z21), 35 (Z35) and 60 (Z60), excluding piston, tip and tilt mode. The AO intensity enhancement (AO on/AO off) was then analysed with respect to the distance (lateral and axial) from the point of correction (somata where AO measurement was performed) to estimate the AO isoplanatic patch size (FOV over which the intensity enhancement (AO on/AO off > 1). In total 6 datasets were analysed (total 311 neurons, from 6 image trials in 3 mice). Corresponding aberrations and depth of AO measurements are displayed in Extended Data Fig. 8. **a**, Lateral (Top, xy) and orthogonal (Bottom, xz) view of maximum intensity projection image of example data-set at 383-683μm depth across 360×360μm lateral FOV acquired without (AO off) and full (AO on Z35) aberration correction. Scale bar 20μm. **b**, Instance segmentation of neuron somata in (a). **c**, Relative position of centroids of all segmented somata (311 neurons) pooled from 6 datasets with respect to point of correction (x=0, y=0, z=0). **d**, Spatial projection of somata (all 6 data-sets) with respect to lateral (R_xy_) and axial (R_z_) distance from point of correction (R_xy_ =0, R_z_ =0). Colour coding represents Intensity enhancement (AO on/ AO off) where red colour is >1, and green <1**. e**, Lateral and axial data from (c) grouped into distance increments of 50 µm. For lateral analysis only somata with R_z_ < 50 µm and accordingly for the axial analysis only somata with R_xy_< 50 µm where analyzed. Boxplot shows median value as red line. Boundaries of box represent upper 75^th^ and lower 25^th^ percentile, whiskers correspond to +/–2.7σ and 99.3 percent coverage. The region where intensity enhancement (AO on/AO off) is <1 is indicated by red box. Analysis shows that the isoplanatic patch (AO on/AO off > 1) is larger in the axial compared to the lateral dimension. Summary plot for Z35 is included in Fig. 2h. Source data

**Extended Data Fig. 7:**
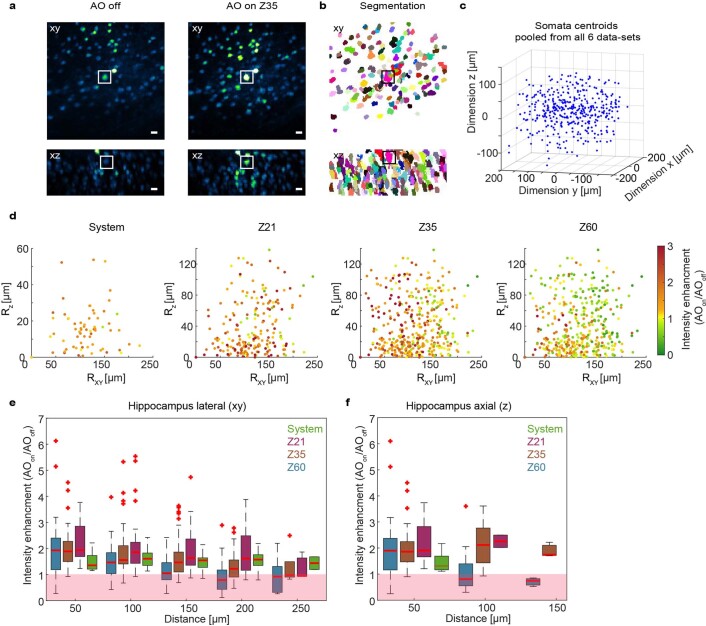
Spatial AO enhancement analysis for local aberration correction in-vivo in Thy1-GFP mouse hippocampus. Aberration measurement was performed for four different conditions referred to: system aberration correction (‘AO system’) and full AO aberration correction inside cortex including Zernike modes up to order 21, (Z21), 35 (Z35) and 60 (Z60), excluding piston, tip and tilt mode. The AO intensity enhancement (AO on/AO off) was then analysed with respect to the distance (lateral and axial) from the point of correction (somata where AO measurement was performed) to estimate the AO isoplanatic patch size (FOV over which the intensity enhancement (AO on/AO off > 1). In total 6 datasets were analysed (total 396 neurons, from 6 image trials in 3 mice). Corresponding aberrations and depth of AO measurements are displayed in Extended Data Fig. 8. **a**, Lateral (Top, xy) and orthogonal (Bottom, xz) view of maximum intensity projection image of example dataset at 1086-1206μm depth across 360×360μm lateral FOV acquired without (AO off) and full (AO on Z35) aberration correction. Scale bar 20μm. **b**, Instance segmentation of neuron somata in (a). **c**, Relative position of centroids of all segmented somata (396 neurons) pooled from 6 datasets with respect to point of correction (x=0, y=0, z=0). **d**, Spatial projection of somata (all 6 datasets) with respect to lateral (R_xy_) and axial (R_z_) distance from point of correction (R_xy_ =0, R_z_ =0). Colour coding represents intensity enhancement (AO on/ AO off) where red colour is >1, and green <1**. e**, Lateral and axial data from (c) grouped into distance increments of 50 µm. For lateral analysis only somata with R_z_ < 50 µm and accordingly for the axial analysis only somata with R_xy_< 50 µm where analyzed. Boxplot shows median value as red line. Boundaries of box represent upper 75^th^ and lower 25^th^ percentile, whiskers correspond to +/–2.7σ and 99.3 percent coverage. The region where intensity enhancement (AO on/AO off) is <1 is indicated by red box. Analysis shows that the isoplanatic patch (AO on/AO off > 1) is larger in the axial compared to the lateral dimension. Summary plot for Z35 is included in Fig. 2h. Source data

Our capability to sense aberrations and perform AO correction throughout entire cortical and subcortical regions allowed us to analyze spatial and depth-associated differences in aberrations and to characterize the isoplanatic patch (that is, the FOV over which a given correction yields an improvement in image signal). We found that the isoplanatic patch was substantially smaller in the lateral than axial dimension (Fig. 2h) and generally decreased with image depth between the cortex (Extended Data Fig. 6) and hippocampus (Extended Data Fig. 7). This suggests that (tissue) aberrations predominantly change laterally across the FOV, and accumulate less with depth, presumably because axial translation of the focus produces more common path aberrations than lateral displacements.

We further performed AO correction with varying Zernike (Z) mode numbers up to Z21, Z35 and Z60, respectively, and found that low-order correction (Z21) led to the highest mean signal enhancement across a large (360 × 360 µm) FOV. In contrast, higher-order Zernike mode correction (Z60) led to the highest local signal enhancement but overall reduced isoplanatic patch size, both laterally as well as axially (Extended Data Figs. 6e,f and 7e,f). Here we found that corrections up to Z35 represented the optimal trade-off between signal enhancement and isoplanatic patch size when imaging in deeper (>500 µm) brain regions over larger axial ranges (Supplementary Video 6). Our findings are relevant for choosing optimal AO strategies for a given imaging condition or desired performance with respect to FOV, depth, resolution enhancement and AO optimization speed. Being able to individually control Zernike modes and to quickly adjust to the desired correction order and number of Zernike modes is an advantage of our modal-based, sensorless AO strategy. Furthermore, this allows the total number of AO measurements to be optimized in terms of overall speed.

**Extended Data Fig. 8:**
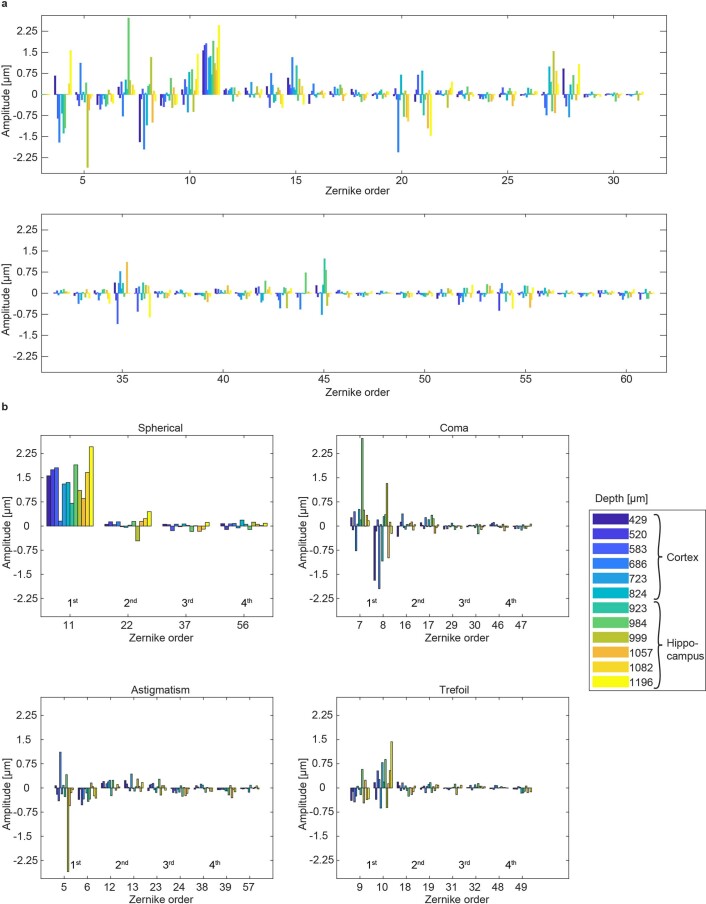
Zernike polynomials used for aberration correction at various depth in cortex and hippocampus. **a**, All modulated Zernike modes 4-61. **b**, Zernike modes grouped by aberrations class: spherical, coma, astigmatism and trefoil. Same data set as Extended Data Figs. 6&7. Source data

**Extended Data Fig. 9:**
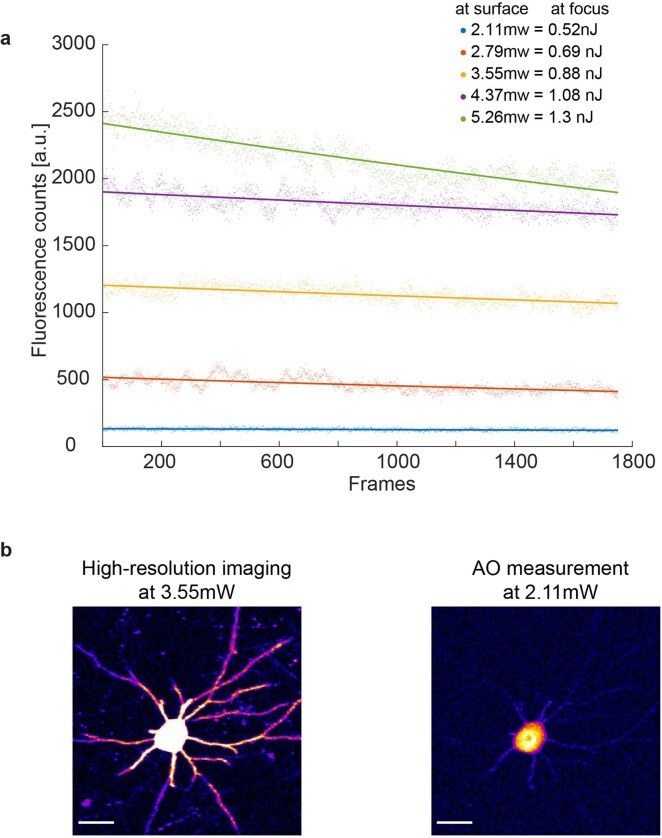
Bleaching levels for different excitation powers and estimated focal energies of imaging conditions utilized in-vivo using Thy1-GFP mouse at 630 μm depth. **a**, Fluorescence signal level versus number of imaged frames for different surface power levels. The corresponding estimated focal energies were estimated based on attenuation length (EAL = 275μm, Extended Data Fig. 2). **b**, Images acquired with (Left) 3.55 mW and (Right) 2.1 mW surface power, as utilized during typical high-resolution imaging and AO measurements, respectively, corresponding to yellow and blue bleaching curves in (a). From (a) both imaging conditions show negligible bleaching effects and are below <1nJ pulse energy, thus well within the previously established safe pulse energy regime^7^. Scale bar 20μm. Representative result of (n=3) experiments. Source data

In general, we found that overall aberration magnitude and r.m.s.e were lower in the cortex than the hippocampus (Fig. 2i); however, individual Zernike mode amplitudes did not display a clear correlation with tissue depth or aberration class (Extended Data Fig. 8). Finally, we characterized bleaching rates at the power levels and pulse energies used during AO measurements and imaging, and found that even at the highest power levels used for high-resolution spine imaging, only negligible bleaching was observed (Extended Data Fig. 9).

As a further demonstration, we applied our method to thinned skull and full, intact skull preparations, which are optically challenging because of considerable bone-induced aberrations that normally restrict high-resolution imaging to the first hundred micrometers^6,37^. While we did observe improvements in image quality in these conditions to a maximum depth of 700 µm (Supplementary Figs. 7 and 8), the signal enhancement was restricted to small image regions around the point of AO measurement, probably because of strong differences in aberrations (small isoplanatic patch size) across the FOV.

### Applications to astrocyte calcium imaging

Next, we explored the capability of our 3P-AO scope for an even more challenging application of multiphoton microscopy, namely in studying the physiology of glial cells that are notorious for low fluorescence in their fine processes and high photosensitivity^38^. Similar to neurons, astrocytes, the most abundant glial cell-type in the brain, constitute a diverse population of cells with morphological and functional heterogeneity across different brain areas^39^.

**Fig. 3:**
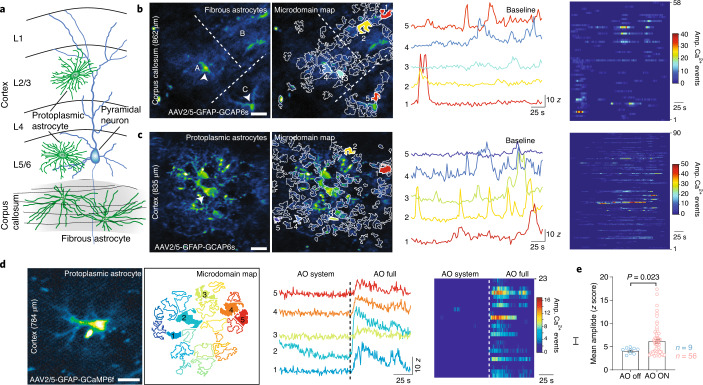
3P-AO enables structural and functional imaging of astrocytes in vivo. **a**, Illustration of protoplasmic and fibrous astrocytes that reside in the gray and white matter, respectively. **b**, White matter astrocytic Ca^2+^-imaging. Left, median intensity time-series projection image (pseudocolored) of three fibrous astrocytes (A, B, C) in the corpus callosum (at a depth of 862 µm). Arrowheads show soma of astrocytes. Map of all active microdomains at baseline overlaid on median intensity projected image of astrocyte. Center, intensity versus time traces for five microdomains (corresponding to colors in left panel), showing characteristics of Ca^2+^ transients. Right, raster plots displaying duration and intensity of Ca^2+^ transients of all microdomains. **c**, Gray matter astrocytic Ca^2+^ imaging. Left, median intensity time-series projection image (pseudocolored) of two protoplasmic astrocytes in the layer 6 of the visual cortex (at a depth of 835 µm). Map of all active microdomains at baseline overlaid on median intensity projected image of astrocyte. Center, intensity versus time traces for five microdomains (corresponding to colors in the left panel), showing characteristics of Ca^2+^ transients. Right, raster plots displaying duration and intensity of Ca^2+^ transients of all microdomains. **d**, AO-enhanced astrocytic Ca^2+^-imaging. Left, median intensity time-series projection image (pseudocolored) of protoplasmic astrocyte in the cortex (at 784 µm deep). Map of active microdomains at baseline. Center, intensity versus time traces for five microdomains (corresponding to colors in left panel), showing characteristics of Ca^2+^ transients without AO (AO off) and full AO (AO on) correction. Right, raster plots displaying duration and intensity of Ca^2+^ transients without AO and full AO correction. **e**, Graph showing mean amplitude (*Z* score/SNR) for microdomain Ca^2+^ transients without AO and full AO correction; *P* value based on a nonparametric Kolmogorov–Smirnov unpaired two-sided *t*-test. Error bars denote s.e.m. Representative datasets shown from *n* = 3 mice, and *n* = 5 L5/6 protoplasmic cells (**c**), *n* = 4 fibrous cells (**b**) and *n* = 3 cells with AO (**d**). Scale bar in **b**–**d**, 20 µm. Source data

The two most prominent astrocyte populations, protoplasmic astrocytes and fibrous astrocytes, reside in gray- and white-matter areas, respectively (Fig. 3a). While protoplasmic astrocytes have been extensively investigated noninvasively in vivo in the upper cortical layers using common two-photon methods^40^, little is known about the Ca^2+^ signals of protoplasmic astrocytes and fibrous astrocytes located in deeper brain areas. The main hurdle in studying fibrous astrocytes has been their location among highly scattering myelinated fiber tracts deep within the brain (>800 µm) and the lack of a genetic tool-box to target these cells. Moreover, astrocytes are highly amenable to photoactivation, and prolonged exposure to high laser power can severely affect their physiology^38,40–43^.

To further demonstrate our technical advancements, we used an adeno-associated virus-(AAV-) based viral approach to express the genetically encoded Ca^2+^ sensor GCaMP6f specifically in astrocytes, and used our 3P-AO microscope to in vivo record Ca^2+^ transients in fibrous astrocytes in the corpus callosum (Fig. 3b) and in deep-layer (layer V-VI) protoplasmic astrocytes (Fig. 3c, Supplementary Video 7 and Supplementary Fig. 9). Here, the use of AO was crucial to achieve high signal and SBR even under low laser excitation power, and enabled the detection of more active microdomains compared to experiments without aberration correction (Fig. 3d,e and Supplementary Videos 8–10). We note that the overall time of AO optimization was fast (<500 ms per independent Zernike mode) compared to Ca^2+^ dynamics lasting for several seconds in the astrocyte soma, which allowed us to use indirect AO on structures with functionally varying signal levels. We resolved Ca^2+^ transients in individual microdomains, which are linked to synaptic activity, and yielded high SNRs even at low power levels. Our approach enabled the discovery that, although fibrous astrocytes reside in the white matter and do not tend to form ‘regular’ synaptic contacts as seen in protoplasmic astrocytes, they still exhibit microdomain-like Ca^2+^ transients.

## Discussion

In summary, by combining 3PM with active aberration correction and ECG gating, we advanced near-diffraction-limited in vivo brain imaging in mice to a depth of 1.4 mm. We note that with our AO strategy we obtained exceptionally high axial correction range, which so far was only thought to be achievable in more elaborate, multi-conjugate AO approaches^44–46^. This permits AO enhancements to be achieved in regions far away from the site of AO measurements and optimization. This can be advantageous, since it allows the use of cell soma for AO measurements that reside in different axial planes or cortical layers. Here, cell soma are more reliable targets for AO optimization, and allow the use of much lower excitation power compared to when dendrites or spines would need to be imaged for AO measurements.

While the acquisition speed in (deep) 3PM is in general limited to a few frames per second owing to limitations associated with laser power, pulse energy and sample physiology^20^, our independent modal-based optimization is sufficiently fast to be used on samples with slowly varying signal levels, including Ca^2+^ signals in astrocytes. This is an advantage of our method compared to other approaches^18,36^ that require fluorescence levels to stay constant over much longer time frames. Compared to direct AO methods, indirect AO optimization, since it is applied to the excitation light, is not sensitive to differences in (tissue) aberrations that might exist between the excitation and signal emission wavelengths, and not depth limited by the wavelength of the (shorter) fluorescence light.

On the application side, our methods allow investigation of biological questions such as layer-specific synaptic remodeling, which was previously restricted to the first three layers of the brain, required removal of overlying brain tissue^9^ or insertion of invasive, optical probes^47^. Furthermore, we showed the capability of our method to uncover the distinct physiology of astrocytes across different cortical layers and of a previously understudied population of subcortical white matter astrocytes. While we ensured that average laser power and focal pulse energies were well below any previously established damage and heating thresholds for neural imaging, we highlight the need for appropriate control experiments in biological applications to ensure minimal photodamage, phototoxicity or photoactivation. Apart from our chosen demonstrations, we believe that our intravital 3P-AO technology can be extended to other tissues and across various model organisms to investigate cellular structure and physiology (and pathophysiology) with minimal invasiveness. In the future, wavefront-shaping approaches^16,17^, which can correct for both multiple scattering and low-order aberrations simultaneously, will have great potential when combined with 3PM to maintain diffraction-limited performance and effective power delivery to the focus at even greater depths.

## Methods

### Excitation source

The 3P excitation source was a wavelength-tunable noncollinear optical parametric amplifier (Spectra Physics) pumped by a regenerative amplifier (Spirit, Spectra Physics) operated at 1,300 nm and 400 kHz. At this wavelength, the laser system delivered an output power of 470 mW with a pulse width of 32 fs. To compensate for group delay dispersion of the microscope optical components, we implemented a homebuilt single-prism pulse compressor^48^, which consisted of a N-SF11, 40 mm prism (PS855, Thorlabs) and two gold roof mirrors (HRS1015-M01, Thorlabs). For imaging in the hippocampus an alternative laser system was used (White Dwarf WD-1300, Class5Photonics), which was operated at 1,300 nm and 500 kHz, delivering 1 W of output power. Dispersion compensation was achieved via dispersive mirrors, which was included in the laser system. With these pulse compressors, roughly 45 fs (Spectra Physics) or 60 fs (Class5Photonics) pulses (deconvolved with a sech^2^ pulse shape) could be recovered at the objective focal plane, as measured by an intensity autocorrelator (Carpe, APE GmbH). The excitation power was modulated with a Pockels cell (360-40 LTA, ConOptics).

### Custom-built AO multiphoton microscope

A schematic of the microscope set-up is shown in Extended Data Fig. 1. For AO correction the DM (for details, see below) was conjugated to the back focal plane of a high-NA objective (NA 1.05, ×25, XLPLN25XWMP2, Olympus) via a pair of relay lenses (for DM-Multi-3.5 (Boston Micromachines); L1, L2, 47-380, Edmund Optics; for DM97-15 (Alpao); L1, AC508-250-C; L2, AC254-75-C, Thorlabs) to galvanometers (GVS002, Thorlabs), which were conjugated to each other with a relay system of two paired lenses (lens L3, 47-380; Edmund Optics; L4, LE4950, Thorlabs). The galvometers, scan lens, tube lens and objective back focal plane were conjugated in a ‘4f’-configuration. The scan lens was composed of 2-inch lenses (AC508-100-C, Thorlabs) in Ploessl configuration and a broad-bandwidth tube lens (TL200-2P2, Thorlabs). The microscope head and detection unit were based on a Movable Objective Microscope (Sutter Instrument Company), and three-dimensional (3D) translation of the objective was achieved with an electronic micromanipulator translation stage (MP-285). The fluorescent signal was collected by the same objective, separated from the excitation path with a long-pass dichroic beam splitter (D1 FF76-875, AHF) and filtered with a near-infrared laser blocking filter (FF01-940/SP, Semrock). The THG and fluorescent signals were separated with a dichroic beam splitter (D2 T480lpxr, AHF), cleaned with optical filters (F1, FF01-432/36-30-D, Semrock; F2, ET525/70m-2p, AHF), respectively, and detected by a photomultiplier tube (Hamamatsu, H7422-40), amplified and low-pass filtered by a transimpedance amplifier (3.5 MHz bandwidth, 10^5^× gain, Femto DHCPA-200). The amplified voltage signals were digitized at a sampling rate of 80 MHz by an A/D conversion module comprising a digitizer adapter (NI-5734, National Instruments) and an FPGA card (PXI-7961R, National Instruments). ScanImage 2016a (Vidrio Technologies^49^) running on MATLAB 2016a (MathWorks) was used to control image acquisition and custom modified to enable synchronization of the image acquisition to the laser pulse repetition rate, integrated by the FPGA to realize a scheme in which each voxel is illuminated by a single or constant multiple of laser pulses. Synchronization to the laser repetition rate also allowed the introduction of a signal sampling window (125 ns was found to be optimal) that ignores noisy samples where no signal was present, thus improving the SNR of the collected data.

### DM calibration

For active wavefront modulation, two different continuous membrane DMs were used: a DM-Multi-3.5 (Boston Micromachines) and the DM97-15 (Alpao) with 140 and 97 actuators, respectively. Calibration and generation of a Zernike-to-Command control matrix (Z2C) was performed with a homebuilt Michelson-interferometer^50^ to calibrate the Boston-DM while the Alpao-DM was inhouse calibrated with a homebuilt Shack–Hartmann wavefront sensor, consisting of a sCMOS camera (C11440-22CU, ORCA Flash4.0, Hamamatsu) with a 20 × 20 microlens array (18-00197, SUSS MicroOptics) and custom-written control software (additionally the Alpao-DM was precalibrated in the factory). Continuous membrane DMs have nonlinear response behavior that results in the Z2C matrix being linear only around the empirically chosen amplitude range used for calibration. To ensure a reliable operation even outside this range, we characterized the effect of Zernike modes on the axial displacement of the PSF. Axial shifts of the image plane are problematic in modal-based AO correction as this can lead to changes of the metric value due to content change of the image rather due to change of the excitation PSF and hence can lead to sample dependent biases^51^. This may corrupt the optimization procedure and prevent convergence to an optimal solution. We observed that independent of the calibration procedure, both DMs showed cross-talk between spherical modes and defocus. Therefore, a look-up table was generated for the Z2C matrix after calibration that characterized the respective focal shift for the first, second and third-order spherical mode. For moderate mode amplitudes (roughly 60% of maximum r.m.s.e value) mode coupling was linear, therefore the focus shift for spherical modes could be compensated by applying the opposite focal shift directly to the DM using the Zernike defocus mode (Supplementary Fig. 2). However, due to nonlinear mode coupling this focus shift correction failed for large amplitudes. Hence, for large amplitude aberrations the axial focal shift of spherical modes was compensated by moving the microscope translation stage along the axial dimension. This approach was integrated into our inhouse custom-written indirect AO software (described below) and the axial translation was based on the generated focal shifts look-up table.

### Modal-based indirect wavefront optimization

For wavefront optimization, a user-defined number (*N*) of Zernike modes were corrected independently and the DM wavefront was updated sequentially after optimization of each mode. For each mode, nine amplitude changes were applied during the first optimization round and five amplitude modulations during subsequent iterations. As a quality metric for the wavefront optimization we chose the mean intensity of user-defined structures of interest such as cell soma, as this showed the most robust performance in low SBR conditions. Furthermore, we found cell soma to be less prone to sample dependent biases^51^ and because of their brightness allowed using exceptionally low power during the AO routine. During optimization, the structures of interest were segmented and only the segmented image was analyzed. Segmentation was achieved by intensity thresholding the median filtered (3 × 3 pixel) image. Zernike modes were modulated in the following order: first spherical, second coma and third astigmatism followed by other modes. This modulation scheme was chosen because spherical and coma aberrations are common and known to have large amplitudes in in vivo cranial window imaging as they result from refractive index mismatches between layers and tilted surfaces, respectively^52^. In general, two to three AO iterations were performed before convergence of the optimization metric was observed. Hence, in total 9 *N* + (*i* − 1) × 5*N* or (*i* × 5*N*) measurements were performed (*i*, number of iterations) to find the optimal wavefront. Normally, tip/tilt and defocus modes were excluded from the correction scheme, however, at large depths (>1,000 µm) the defocus mode was included into the correction scheme to reposition the focal plane onto the object of interest after every iteration as vital functions of the animal could lead to translational brain motion and hence sample dependent biases. This can lead to an elongation of the PSF toward the brightest structure if it is not centered at the initial focal plane^51^.

### Typical parameters for 3P-AO optimization

For indirect modal-based wavefront optimization the focal plane was positioned in the center of the brightest structure in the region of interest. For in vivo experiments, we chose neurons (Thy1-GFP mice) or astrocyte (expressing GCaMP6f) somata for indirect AO optimization. Typically, a 40 × 40 μm FOV was scanned with a 64 × 64 pixel resolution, resulting in a roughly 25 Hz acquisition rate during optimization. Independent mode optimization was essential for aberration measurement on dynamic astrocyte calcium signals. With an independent mode optimization scheme the fluorescent signal only needs to be stable over 5–9 amplitude modulations. These data could be acquired within around 250–450 ms, which is at least one order of magnitude faster than somatic astrocyte GCaMP dynamics (about 8.0 s, ref. ^53^). For cranial window preparations, typically 21–60 Zernike modes, excluding tip/tilt mode, were used for aberration correction and 2–3 iterations were performed. Hence, a total of 210–900 measurements were required for aberration measurement that resulted in an overall fluorescence acquisition time of 9–36 s at a 25 Hz frame rate. Acquisition parameters for AO optimization and imaging are summarized in Supplementary Table 2. To minimize brain heating and photobleaching during AO optimization, the excitation power was reduced substantially, approximately by 50%, so that only a dim signal of the large somata was visible. Using our measured parameters for the light attenuation *l*_z_ at 1,300 nm in brain tissue (*l*_z_ = 270 µm, cortex), *l*_z_ = 101 µm (corpus callosum) and *l*_z_ = 250 µm (hippocampus) the estimated focal energy *E*_f_ was roughly 1 nJ (*E*_f_ = *E*_o_ × exp(−*z*/*l*_z_), where *z* is depth from the surface) during wavefront optimization, which is within the established physiological pulse energy regime^7^ (Extended Data Fig. 2). We further characterized bleaching rates at the used power levels and pulse energies at depth, and found that even at highest pulse energies used (that is, roughly 0.9 nJ, corresponding to 3.55 mW (surface) at 630 µm deep), negligible bleaching (<10% fall off within 1,750 frames) was observed (Extended Data Fig. 9). Although we used physiological power and pulse energy regimes, aberration measurements were performed on a different astrocyte within the proximity of the astrocyte of interest used for functional recordings, to ensure physiological responses during the imaging period. Furthermore, to prevent experimental biases and activation of astrocytes during recording the order of AO correction (first AO off followed by AO on, or vice versa) was chosen randomly (Supplementary Videos 8–10). For structural imaging in Thy-1-EGFP mice, the wavefront correction and imaging was performed on the same cell somata. Furthermore, to increase the objective transmission at a greater depth, the effective illumination NA was reduced (NA of roughly 0.8), yielding slightly decreased resolution (Fig. 2g).

To minimize aberrations before AO optimization, the correction collar of the objective was adjusted to compensate for the refractive index mismatch of the cranial window. Furthermore, the cranial window was aligned orthogonal to the optical axis using an objective-like alignment tool that enabled alignment within <1° accuracy. The alignment tool was based on overlaying the reflection from the cranial window to a reference reflection of a surface perpendicular to the optical axis at >2 m distance.

### ECG gating

#### Software development

ScanImage was custom modified to acquire images gated in real time to the physiological parameters of the mouse, such as breath and heart rate, which were monitored by a dedicated physiological monitoring system (ST2 75-1500, Harvard Apparatus). The ECG signal triggered a transistor–transistor logic (TTL) pause gate (generation of the gate is described below), which was used to modify the system’s laser clock that was fed into the FPGA through an auxiliary I/O connector block (SCB-19, National Instruments). When the state of the pause gate was set to high, this effectively halted the image acquisition task and the galvometers stopped at their current position. During this period, the Pockels cell was synchronously turned off via an external circuit-based AND gate to prevent extensive light exposure of the sample. Scanning continued once the pause gate was reset to low.

### Hardware development

The ECG-triggered TTL pause gate was developed on an FPGA System-on-Chip platform (ZYBO Z7 ZYNQ-7010, Digilent) using the Verilog programming language on Vivado 2018. First, the analog ECG signal was scaled to the FPGA input range (0–1 V), using an operational amplifier to reduce the peak-to-peak amplitude of the signal and centering it at 0.5 V. Next, the ECG signal R wave peaks (the local highest amplitude in the ECG signals) of the cardiac cycle was taken as the base point to generate the pause gate. The status of the TTL pause gate was set to low within the time interval (*t*_p_ − Δ*t*_pre_, *t*_p_ + Δ*t*_post_), where the R wave peak occurred at time *t*_p_ (Supplementary Fig. 1a). The parameters Δ*t*_pre_ and Δ*t*_post_ were manually adjustable in accordance with suppression of the motion artifacts in the images. In our experiments, Δ*t*_pre_ and Δ*t*_post_ were set to 30 and 20% of the ECG-cycle period, resulting in an imaging duty cycle of 50%. In practice, it is hard to predict the exact occurrence time of the next R wave peak. Therefore, we continuously analyzed the last ten heartbeat cycles to predict the next R wave peak in real time. Furthermore, we developed an auto-tuning threshold algorithm that adapted the threshold levels to changing DC levels of the ECG signal.

### Animals and ethics statement

This work followed the European Communities Council Directive (2010/63/EU) to minimize animal pain and discomfort. All procedures described in this paper were approved by EMBL’s committee for animal welfare and institutional animal care and use, under protocol number RP170001. Experiments were performed on male and female, 7–24-week-old C57Bl6/j or homozygous Thy1–EGFP-M (Jax no. 007788) transgenic mice from the EMBL Heidelberg core colonies. During the course of the study, mice were housed in groups of 1–5 in makrolon type 2L or 3H cages, in ventilated racks at room temperature and 50% humidity while kept in a 12/12 h light/dark cycle. Food and water were available ad libitum.

### Stereotaxic viral vector delivery and cranial window implantation

For astrocyte-specific expression of GCaMP6f, the gfaABC1D-cyto-GCaMP6f expression construct (Addgene, catalog no. 52925) was subcloned into the AAV vector. rAAV serotypes 1 and 2 viral particles were generated as described previously^54^ and purified by AVB Sepharose affinity chromatography^55^ after titration with real-time PCR (each titer 1.0–6.0 × 10^12^ viral genomes per ml; TaqMan Assay, Applied Biosystems). For stereotaxic injection of rAAVs and the cranial window implantation, mice were anesthetized with isoflurane vapor mixed with O_2_ (5% for induction and 1–1.5% for maintenance). Anesthetized mice were subcutaneously injected with 1% xylocain (AstraZeneca) under the scalp as preincisional local anesthesia and placed in a stereotaxic frame (David Kopf Instruments, model 963). The skin and periosteum over the dorsal cranium were removed with fine forceps and scissors to expose the bone. A 4-mm diameter circular craniectomy was made over the visual cortex using a dental drill (Microtorque, Harvard Apparatus), centered at 2.5 mm posterior and 2.5 mm lateral to Bregma. Close care was taken to preserve the integrity of the dura and avoid bleeding. rAAV injections were performed at the center of the craniectomy using glass pipettes lowered to depths of 400, 800 and 1,200 μm, using a syringe at a rate of roughly 4 μl h^−1^. Roughly 300 nl were injected per spot. After injection, a round 4 mm coverslip (around 170 μm thick, disinfected with 70% ethanol) was placed over the craniectomy with a drop of saline between the glass and the dura. The craniectomy and cranial window were sealed using acrylic dental cement (Hager Werken Cyano Fast and Paladur acrylic powder), and a customized headbar was cemented to the skull for head fixation under the microscope. The skin wound around the surgical area was also closed with dental acrylic. After surgery, mice received pain relief (Metacam, Boehringer Ingelheim) and antibiotic (Baytril, Bayer) injections (subcutaneous, 0.1 and 0.5 mg ml^−1^, respectively, dosed 10 μl g^−1^ body weight). Mice were single housed after cranial window implantation and had a recovery period of at least 3 weeks before further experiments, for GCaMP6f expression and to resolve the inflammation associated with this surgery^56^.

### Thinned skull and brain slice preparation

Skull thinning followed the same general steps as the cranial window implantation procedure described above. Once the skull was exposed, a round approximately 3 mm area centered at 2.5 mm posterior and 2.5 mm lateral to Bregma was thinned using a dental drill. The thinned area was deepened a few micrometers beyond the trabecular part of the bone and the thinned surface was leveled out with polishing dental drill bits, leaving roughly 25–30 μm of remaining bone tissue. The thickness of the thinned area was estimated by visualization of blood vessels through the progressively more transparent thinned skull.

Brain slices were generated by transcardially perfusing the animal with phosphate-buffered saline (PBS) followed by 4% paraformaldehyde to fixate the brain. Subsequently, the perfused brain was isolated and post-fixed in 4% paraformaldehyde for 24 h at 4 °C before being transferred to saline and stored at 4 °C until 300-μm-thick free-floating coronal slices were cut with a vibratome (Leica VT1200).

Imaging through the intact skull was performed through a glass cranial window implanted over the exposed intact bone. This preparation isolated the exposed bone from the environment. The surgical procedure followed the same general steps as the cranial window implantation procedure described above. Once the dorsal skull bone was exposed, a round 4 mm no. 0 coverslip was placed centered at 2.5 mm posterior and 2.5 mm lateral to Bregma, with a saline drop on the underside to fill the gaps between the glass and skull. The whole assembly and skin wound was sealed and fixed to the surrounding exposed skull with dental cement.

### In vivo deep brain imaging

For in vivo imaging experiments, animals were head-fixed using a customized headbar and complement holder and the cranial window was aligned orthogonal to the optical axis using the method described above. For structural neuronal imaging, Thy1-EGFP mice were anesthetized with isoflurane (2% in oxygen, Harvard Apparatus) while for functional astrocyte imaging mice were kept under low anesthesia (isoflurane 1–1.5% in oxygen). Mice were positioned on a small animal physiological monitoring system (ST2 75-1500, Harvard Apparatus), which allowed maintenance of animal body temperature at 37.5 °C and to monitor physiological parameters, ECG signal and breath rate. During experiments, eyes were covered with eye ointment. Acquisition parameters for structural neuronal imaging and functional astrocyte Ca^2+^ imaging are summarized in Supplementary Tables 1 and 2. Here we note that imaging depth was reported as raw axial translation of the objective. Taking the refractive index difference between the coverslip, immersion media and various brain tissues into account^4^, the actual imaging depth is likely around 5–10% larger than the values reported. In general, the data shown in the figures are representative of several imaging sessions, which were repeated between 2 and 11 times each on 16 separate mice, all of which produced comparable data quality. The numbers of mice are indicated in the figure captions.

### Image postprocessing and analysis

All image data shown represent raw, 3 × 3 or 3 × 3 × 3 median filtered images, as stated in the respective captions and summarized in Supplementary Table 1. To improve visibility of dim structures before AO correction a scaling factor was applied to images (also indicated in the figures). Slow image drifts due to translational brain motion were corrected by template-matching individual image frames. Structural neuronal images from Thy1-eGFP mice were registered by rigid body transformation with StackReg^57^ (ImageJ) while astrocyte time series were registered by translation with TurboReg (ImageJ).

For the spatial analysis of aberrations presented in Extended Data Figs. 6 and 7, the measured aberrations for the different AO conditions (AO off, AO system and AO on for Z21, Z35 and Z60; that is, for correction up to Zernike orders of 21, 35 and 60, respectively, excluding piston, tip and tilt), were analyzed. Aberration correction was performed in the cortex (430–830 μm) and hippocampus (920–1,200 μm) and image stacks for the different AO conditions were recorded over a 360 × 360 μm lateral FOV with varying depths (roughly 300–400 μm in cortex and 100–300 μm in hippocampus). As neuronal somata were used for spatial aberration analysis the axial extend of the FOV in the hippocampus was smaller compared to the cortex, as the CA1 region in the hippocampus only exhibited a small region were somata are located. In total, six datasets from both cortex and hippocampus from three mice were used for analysis.

To spatially analyze the AO intensity enhancement of aberration correction (AO on: AO system, AO Z21, Z35, Z60), the ratio of the mean intensity of each somata within a FOV (AO on/AO off) was calculated and analyzed with respect to the lateral and axial distance from the point of correction (that is, the somata on which the AO measurement was performed). In more detail, we carried out the analysis in the following way: AO Z21 was used as the reference for analysis. Somata were segmented using the open-source machine learning-based software Ilastik^58^. The rest of the analysis was conducted in MATLAB. Object detection of individual somata was achieved with the command ‘bwconncomp’ (generating connected components). Objects that were smaller than 100 pixels were discarded and the centroids of each somata object were determined using ‘regionprops’. To prevent bias, image stacks of all AO conditions were aligned in 3D to the reference stack (AO Z21) with ‘imregister’ (transfformtyp, translation). The mean intensity of the top 20% brightest pixels of the somata object labeled pixels was used to calculate the intensity ratio (AO on/AO off).

### Automated extraction and analysis of astrocyte Ca^2+^ signals

Astrocytes’ Ca^2+^ signals were analyzed using a custom-built MATLAB-based machine learning algorithm called CaSCaDe (calcium signal classification and decoding, for details see ref. ^38^). Since CaSCaDe was designed for the analysis of 2PM data, here we incorporated the following changes to optimize the code for analyzing 3PM data: a 3D bandpass filter of 11 × 11 × 11 pixels was applied to the time-series image, the sum-intensity projected image was binarized using a threshold value of *I*_bg_, the image was smoothed with a 31 × 31 pixels^2^ Gaussian filter and a standard deviation of 3 pixels. To generate a microdomain map, a watershed segmentation with more than two regional maxima was applied, and microdomains with an area of less than 5 × 5 pixels^2^ were considered as noise and discarded. A Ca^2+^ event was considered positive if it had an intensity value of 3 (a.u.) and it spanned more than four frames. A support vector machine was trained to further refine the detection of Ca^2+^ events. Then 75 features were extracted from raw and two smooth intensity profiles obtained using a Gaussian filter with sizes of 11 and 21 frames separately. Support vector machine training was performed using MATLAB on 4,300 fluorescence signals manually annotated as positive or negative.

### Reporting Summary

Further information on research design is available in the Nature Research Reporting Summary linked to this article.

## Online content

Any methods, additional references, Nature Research reporting summaries, source data, extended data, supplementary information, acknowledgements, peer review information; details of author contributions and competing interests; and statements of data and code availability are available at 10.1038/s41592-021-01257-6.

## Supplementary information

Supplementary Information Supplementary Figs. 1–9 and Tables 1 and 2.

Reporting Summary

Supplementary Video 1 SI_video1.

Supplementary Video 2 SI_video2.

Supplementary Video 3 SI_video3.

Supplementary Video 4 SI_video4.

Supplementary Video 5 SI_video9.

Supplementary Video 6 SI_video10.

Supplementary Video 7 SI_video5.

Supplementary Video 8 SI_video6.

Supplementary Video 9 SI_video7.

Supplementary Video 10 SI_video8.

## Data Availability

The main datasets generated and/or analyzed during this study are available either as source data files or at 10.5281/zenodo.5060975. Source data are provided with this paper.

## Source data

Source Data Fig. 1 Source data for Fig. 1
Source Data Fig. 2 Source data for Fig. 2
Source Data Fig. 3 Source data for Fig. 3
Source Data Extended Data Fig. 2 Source data for Extended Data Fig. 2
Source Data Extended Data Fig. 4 Source data for Extended Data Fig. 4
Source Data Extended Data Fig. 5 Source data for Extended Data Fig. 5
Source Data Extended Data Fig. 6 Source data for Extended Data Fig. 6
Source Data Extended Data Fig. 7 Source data for Extended Data Fig. 7
Source Data Extended Data Fig. 8 Source data for Extended Data Fig. 8
Source Data Extended Data Fig. 9 Source data for Extended Data Fig. 9

## Code availability

The indirect AO routines developed in this work for integration into ScanImage^32^ are available at https://github.com/prevedel-lab/AO.git. The FPGA code and electronics developed for the ECG gating are available at https://github.com/prevedel-lab/ECG-gating.git.

## References

[1] Helmchen F, Denk W (2005). Deep tissue two-photon microscopy. Nat. Methods.

[2] Ji N, Freeman J, Smith SL (2016). Technologies for imaging neural activity in large volumes. Nat. Neurosci..

[3] Theer P, Denk W (2006). On the fundamental imaging-depth limit in two-photon microscopy. J. Opt. Soc. Am. A..

[4] Horton NG (2013). In vivo three-photon microscopy of subcortical structures within an intact mouse brain. Nat. Photonics.

[5] Ouzounov DG (2017). In vivo three-photon imaging of activity of GCaMP6-labeled neurons deep in intact mouse brain. Nat. Methods.

[6] Wang T (2018). Three-photon imaging of mouse brain structure and function through the intact skull. Nat. Methods.

[7] Yildirim M, Sugihara H, So PTC, Sur M (2019). Functional imaging of visual cortical layers and subplate in awake mice with optimized three-photon microscopy. Nat. Commun..

[8] Barretto RPJ, Messerschmidt B, Schnitzer MJ (2009). In vivo fluorescence imaging with high-resolution microlenses. Nat. Methods.

[9] Dombeck DA, Harvey CD, Tian L, Looger LL, Tank DW (2010). Functional imaging of hippocampal place cells at cellular resolution during virtual navigation. Nat. Neurosci..

[10] Ji N (2017). Adaptive optical fluorescence microscopy. Nat. Methods.

[11] Booth MJ (2014). Adaptive optical microscopy: the ongoing quest for a perfect image. Light.: Sci. Appl..

[12] Wang K (2015). Direct wavefront sensing for high-resolution in vivo imaging in scattering tissue. Nat. Commun..

[13] Liu R, Li Z, Marvin JS, Kleinfeld D (2019). Direct wavefront sensing enables functional imaging of infragranular axons and spines. Nat. Methods.

[14] Ji N, Sato TR, Betzig E (2012). Characterization and adaptive optical correction of aberrations during in vivo imaging in the mouse cortex. Proc. Natl Acad. Sci. USA.

[15] Galwaduge PT, Kim SH, Grosberg LE, Hillman EMC (2015). Simple wavefront correction framework for two-photon microscopy of in-vivo brain. Biomed. Opt. Express.

[16] Horstmeyer R, Ruan H, Yang C (2015). Guidestar-assisted wavefront-shaping methods for focusing light into biological tissue. Nat. Phot..

[17] Papadopoulos IN, Jouhanneau J-S, Poulet JFA, Judkewitz B (2017). Scattering compensation by focus scanning holographic aberration probing (F-SHARP). Nat. Photonics.

[18] Park J-H, Sun W, Cui M (2015). High-resolution in vivo imaging of mouse brain through the intact skull. Proc. Natl Acad. Sci. USA.

[19] Podgorski K, Ranganathan G (2016). Brain heating induced by near-infrared lasers during multiphoton microscopy. J. Neurophysiol..

[20] Wang T, Xu C (2020). Three-photon neuronal imaging in deep mouse brain. Optica.

[21] Wang T (2020). Quantitative analysis of 1300-nm three-photon calcium imaging in the mouse brain. eLife.

[22] Lee S, Vinegoni C, Sebas M, Weissleder R (2015). Automated motion artifact removal for intravital microscopy, without a priori information. Sci. Rep..

[23] Paukert M, Bergles DE (2012). Reduction of motion artifacts during in vivo two-photon imaging of brain through heartbeat triggered scanning. J. Physiol..

[24] Nimmerjahn A (2005). Resting microglial cells are highly dynamic surveillants of brain parenchyma in vivo. Science.

[25] Laffray S (2011). Adaptive movement compensation for in vivo imaging of fast cellular dynamics within a moving tissue. PLoS ONE.

[26] Chen JL, Pfäffli OA, Voigt FF, Margolis DJ, Helmchen F (2013). Online correction of licking-induced brain motion during two-photon imaging with a tunable lens. J. Physiol..

[27] Griffiths VA (2020). Real-time 3D movement correction for two-photon imaging in behaving animals. Nat. Methods.

[28] Weisenburger S (2019). Volumetric Ca^2+^ imaging in the mouse brain using hybrid multiplexed sculpted light microscopy. Cell.

[29] Booth MJ, Neil MAA, Juskaitis R, Wilson T (2002). Adaptive aberration correction in a confocal microscope. Proc. Natl Acad. Sci. USA.

[30] Débarre D (2009). Image-based adaptive optics for two-photon microscopy. Opt. Lett..

[31] Ji N, Milkie DE, Betzig E (2010). Adaptive optics via pupil segmentation for high-resolution imaging in biological tissues. Nat. Methods.

[32] Planchon TA (2011). Rapid three-dimensional isotropic imaging of living cells using Bessel beam plane illumination. Nat. Methods.

[33] Hu Q (2020). A universal framework for microscope sensorless adaptive optics: generalized aberration representations. APL Photonics.

[34] Sinefeld D, Paudel HP, Ouzounov DG, Bifano TG, Xu C (2015). Adaptive optics in multiphoton microscopy: comparison of two, three and four photon fluorescence. Opt. Express.

[35] Milkie DE, Betzig E, Ji N (2011). Pupil-segmentation-based adaptive optical microscopy with full-pupil illumination. Opt. Lett..

[36] Rodríguez, C. et al. An adaptive optics module for deep tissue multiphoton imaging in vivo. Preprint at *bioRxiv* https://doi.org/2020.11.25.397968 (2020). 10.1038/s41592-021-01279-0 PMC9090585 34608309

[37] Isshiki M, Okabe S (2014). Evaluation of cranial window types for in vivo two-photon imaging of brain microstructures. Microscopy.

[38] Agarwal A (2017). Transient opening of the mitochondrial permeability transition pore induces microdomain calcium transients in astrocyte processes. Neuron.

[39] Khakh BS, Deneen B (2019). The emerging nature of astrocyte diversity. Annu. Rev. Neurosci..

[40] Semyanov A, Henneberger C, Agarwal A (2020). Making sense of astrocytic calcium signals—from acquisition to interpretation. Nat. Rev. Neurosci..

[41] Stobart JL (2018). Cortical circuit activity evokes rapid astrocyte calcium signals on a similar timescale to neurons. Neuron.

[42] Poskanzer KE, Yuste R (2016). Astrocytes regulate cortical state switching in vivo. Proc. Natl Acad. Sci. USA.

[43] Otsu Y (2015). Calcium dynamics in astrocyte processes during neurovascular coupling. Nat. Neurosci..

[44] Park J-H, Kong L, Zhou Y, Cui M (2017). Large field-of-view imaging by multi-pupil adaptive optics. Nat. Methods.

[45] Paudel HP, Taranto J, Mertz J, Bifano T (2015). Axial range of conjugate adaptive optics in two-photon microscopy. Opt. Express.

[46] Mertz J, Paudel H, Bifano TG (2015). Field of view advantage of conjugate adaptive optics in microscopy applications. Appl. Opt..

[47] Pattwell SS (2016). Dynamic changes in neural circuitry during adolescence are associated with persistent attenuation of fear memories. Nat. Commun..

[48] Kong, L. & Cui, M. A high throughput (>90%), large compensation range, single-prism femtosecond pulse compressor. Preprint at *arXiv* https://arxiv.org/abs/1306.5011 (2013).

[49] Pologruto TA, Sabatini BL, Svoboda K (2003). ScanImage: Flexible software for operating laser scanning microscopes. Biomed. Eng. Online.

[50] Antonello, J., Wang, J., He, C., Phillips, M. & Booth, M. Interferometric calibration of a deformable mirror. 10.5281/zenodo.3714951 (Zenodo, 2020).

[51] Champelovier D (2017). Image-based adaptive optics for in vivo imaging in the hippocampus. Sci. Rep..

[52] Turcotte R, Liang Y, Ji N (2017). Adaptive optical versus spherical aberration corrections for in vivo brain imaging. Biomed. Opt. Express.

[53] Paukert M (2014). Norepinephrine controls astroglial responsiveness to local circuit activity. Neuron.

[54] Tang W (2009). Faithful expression of multiple proteins via 2A-peptide self-processing: a versatile and reliable method for manipulating brain circuits. J. Neurosci..

[55] Smith RH, Levy JR, Kotin RM (2009). A simplified baculovirus-AAV expression vector system coupled with one-step affinity purification yields high-titer rAAV stocks from insect cells. Mol. Ther..

[56] Holtmaat A (2009). Long-term, high-resolution imaging in the mouse neocortex through a chronic cranial window. Nat. Protoc..

[57] Thevenaz P, Ruttimann UE, Unser M (1998). A pyramid approach to subpixel registration based on intensity. IEEE Trans. Image Process..

[58] Berg S (2019). ilastik: interactive machine learning for (bio)image analysis. Nat. Methods.

