# Supplementary material for: High-resolution structural and functional deep brain imaging using adaptive optics three-photon microscopy
Source: Nat Methods. 2021 Sep 30;18(10):1253–8. doi: 10.1038/s41592-021-01257-6 (PMC8490155; doi:10.1038/s41592-021-01257-6)
Supplement: Supplementary file 1 — Supplementary Figs. 1–9 and Tables 1 and 2. [file 41592_2021_1257_MOESM1_ESM.pdf]

nature portfolio

https://doi.org/10.1038/s41592-021-01257-6

Supplementary information

High-resolution structural and functional deep brain imaging using adaptive optics three-photon microscopy

In the format provided by the authors and unedited

Supplementary Figures

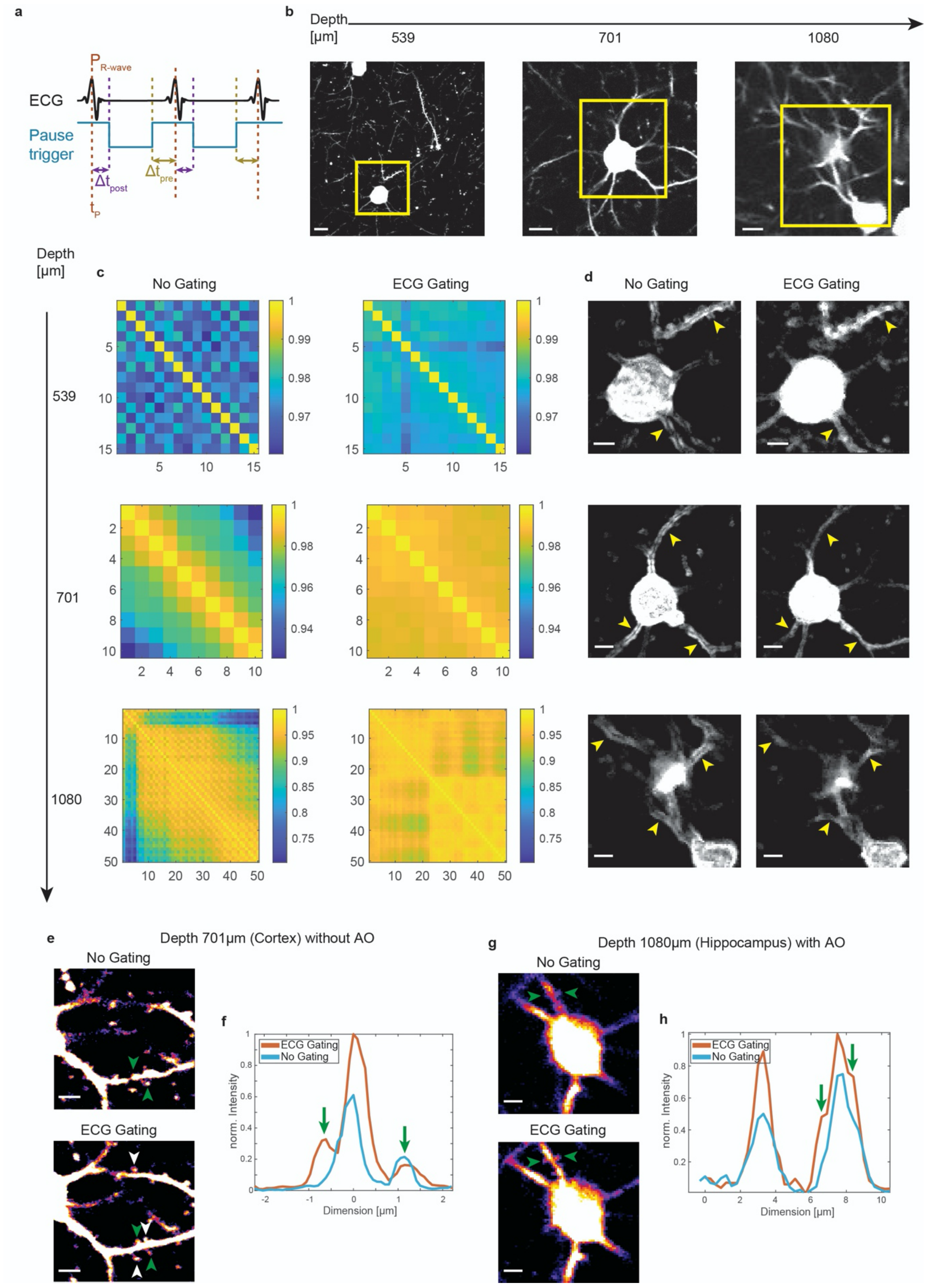

Supplementary Figure 1: **ECG-gating reduces intra-frame motion artefacts in in-vivo three-photon microscopy. (a)** Schematic of the trigger signal (cyan) that gates image acquisition (low during acquisition, high during pause time). ECG R-wave peak (brown) at time

point t_p, capping time Δt_{post} (purple), time difference to the predicted next heart beat, Δt_{pre} (green). **(b)** Average intensity projection of consecutively acquired frames with ECG-gated image acquisition for different depth and acquisition parameters: depth 57µm, 256x256 pixel, pixel dwell 25µs; depth 536µm, 512x512 pixel, pixel dwell 15µs; depth 701µm, 512x512 pixel, pixel dwell 12.5µs; depth 1080µm, 128x128 pixel, pixel dwell 25µs. Scale bar 10µm. **(c)** 2D-cross correlation matrix between pairwise individual frames without (left) and with (right) ECG synchronization. **(d)** Standard deviation projection images (STD) of consecutively acquired image frames with (right) and without (left) ECG gating. Boundaries, indicated by yellow arrows become clearly visible without synchronization while no pronounced boundaries are visible with ECG gating, indicated by yellow arrows. Scale bar 5µm. **(e,g)** Averaged three-photon image of **(e)** 15 consecutively acquired frames (FOV = 34µm) at depth 701µm without adaptive optics correction and **(g)** 10 consecutively acquired frames (FOV = 42µm) at depth 1080µm with adaptive optics correction, and corresponding line plots across spines indicated by green arrows in **(f)** and **(g)**, respectively. Spines, indicated by white and green arrows, become clearly visible with cardiac gated acquisition while these fine structures are blurred out without image synchronization.

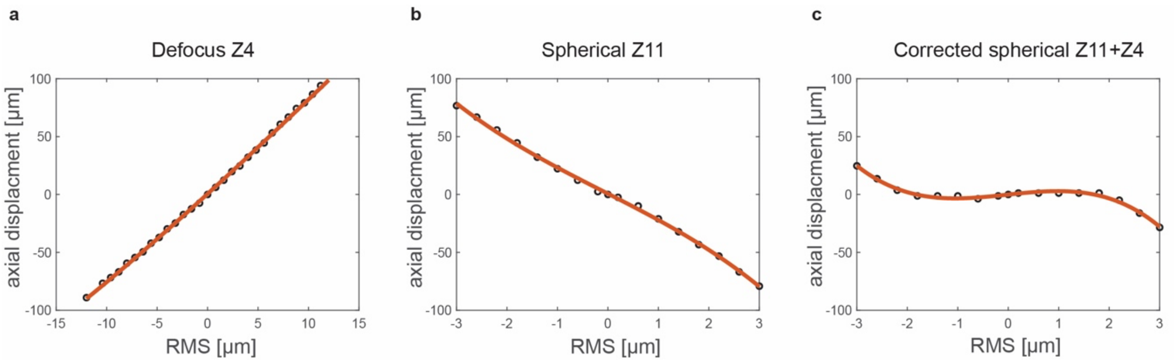

Supplementary Figure 2: **Axial focus shift calibration of DM.** Results shown for DM97-15 (Alpao) and measured with 2µm fluorescent beads for different mode amplitudes for Zernike **(a)** defocus mode Z4 and **(b)** first spherical mode Z11. **(c)** The axial focus shift can partially be compensated (for small amplitudes) by combining the defocus and spherical mode.

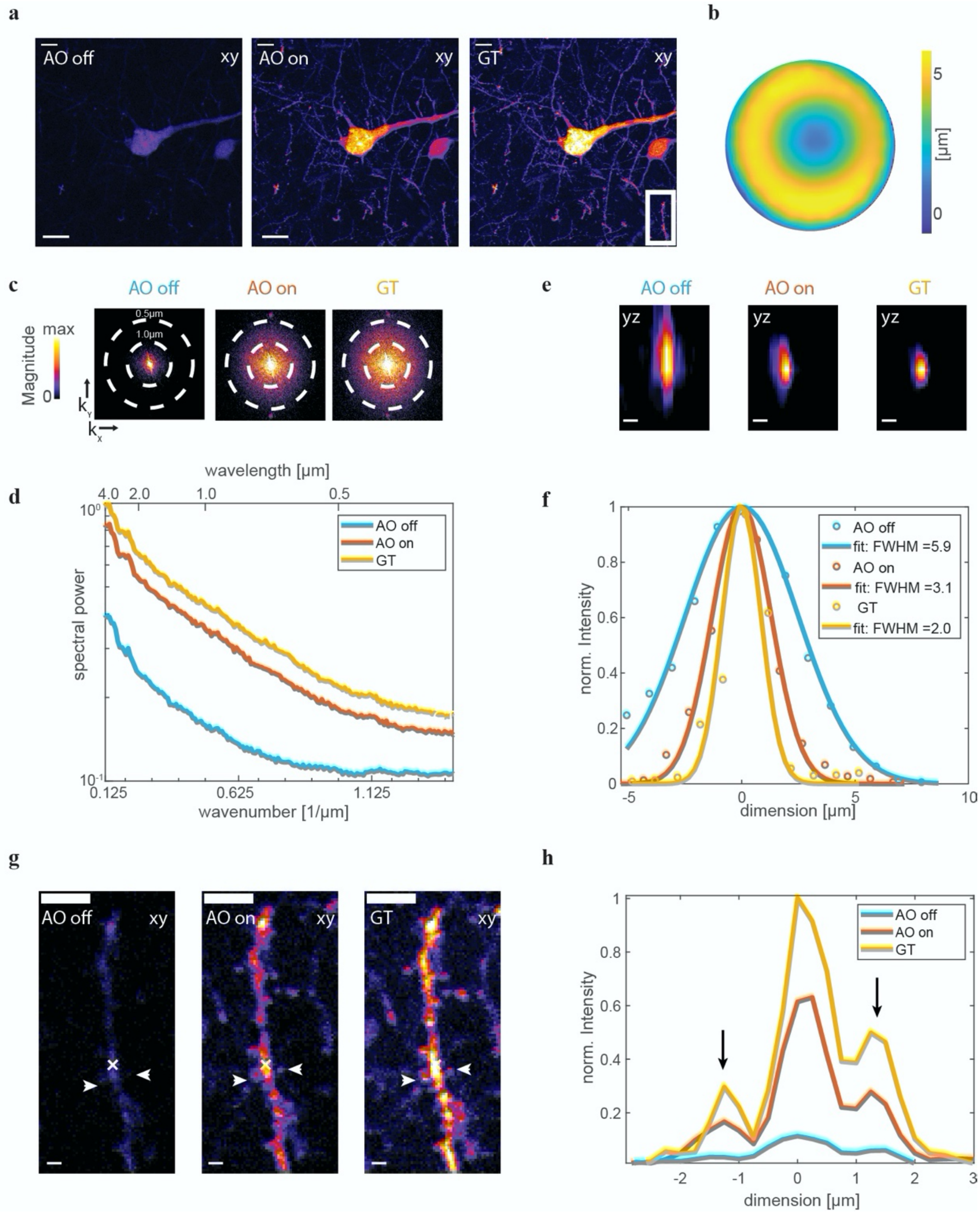

Supplementary Figure 3: Validation of indirect adaptive optics in ex-vivo Thy1-EGFP(M) brain slices. Correction of wavefront aberrations introduced by detuning the objective correction collar. Images were recorded with three different conditions. AO off: detuned correction collar and flat DM; AO on: WF correction and detuned correction collar; GT: optimal correction collar with system aberration correction. **(a)** Maximum Intensity projection image for the three different conditions over 20µm depth. Scale bar 15µm. **(b)** Corrected wavefront (WF) of Alpao DM. **(c)** Spectral power as a function of spatial frequency (wavenumber) for the images in (a). **(d)** Average radial profile of spectral power maps in c). **(e)** Orthogonal view along selected dendrite indicated in g) by white cross. Scale bar 2µm. **(f)** Axial (x,z) intensity profile along dendrite displayed in e) with Gaussian fit to determine the FWHM. **(g)** Magnified views of postsynaptic spines and dendrites corresponding to the boxed region in (a). Scale bar 2µm. **(h)** Lateral (x,y) intensity profile along spines indicated in g) by white arrows. Scale bar in a) and g) 5µm. Scale bar in e) 2µm. This proof-of-principle experiment was performed in one slice (n=1).

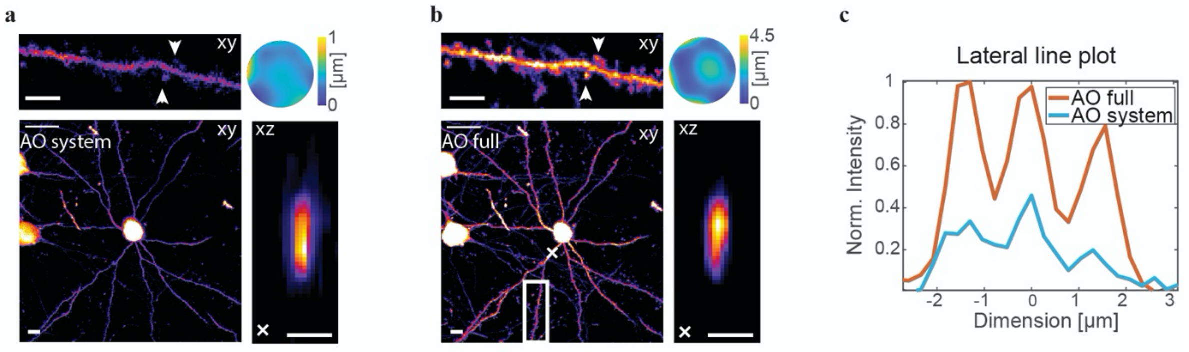

Supplementary Figure 4: Indirect adaptive optics correction and three-photon microscopy imaging of dendrites and spines in layer V at 653μm depth. Images were recorded in Thy1-EGFP(M) mouse cortex in-vivo through a cranial window with ECG-gating and two different conditions. AO system: wavefront correction of system aberrations; AO full: wavefront correction of system and brain tissue aberrations. Maximum intensity projection images at 623-653μm depth with **(a)** system and **(b)** with full adaptive optics correction. White boxes indicate magnified view (shown on top) of spines which clearly become visible with full adaptive optics correction. White cross indicates orthogonal view (xz) along dendrite. Respective wavefronts for aberration correction are displayed in the top corners. Scale bar 5μm. **(c)** Lateral (xy) intensity profile along spines (white dotted line). Representative result of (n=5) experiments.

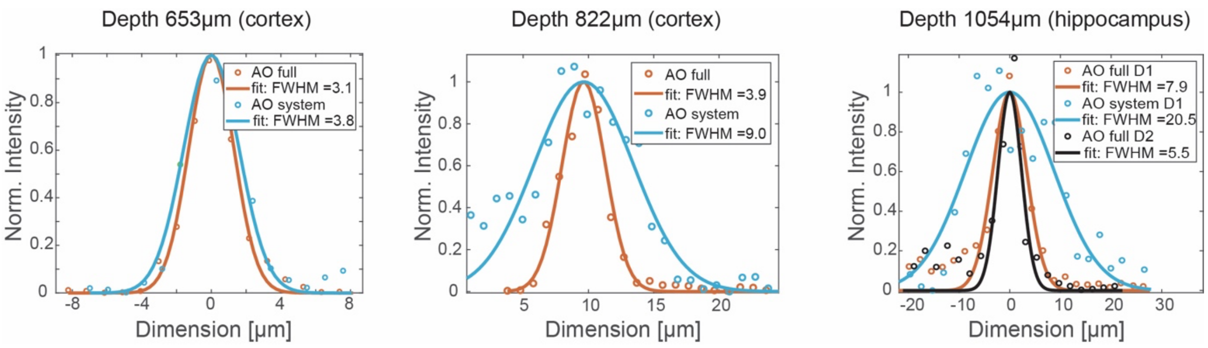

Supplementary Figure 5: Estimated axial resolution of 3P-AO microscopy at depth in the in vivo-mouse brain with system and full adaptive optics correction. Axial (xz/yz) intensity profile at depths of 653μm, 822μm and 1054μm (hippocampus) along dendrite shown in Supplementary Figure 4a,b, Figure 2a, and Supplementary Figure 6b, respectively. Dotted line: experimental data, straight line: Gaussian fit to the data to determine the FWHM which is displayed in the figure legend in micrometer.

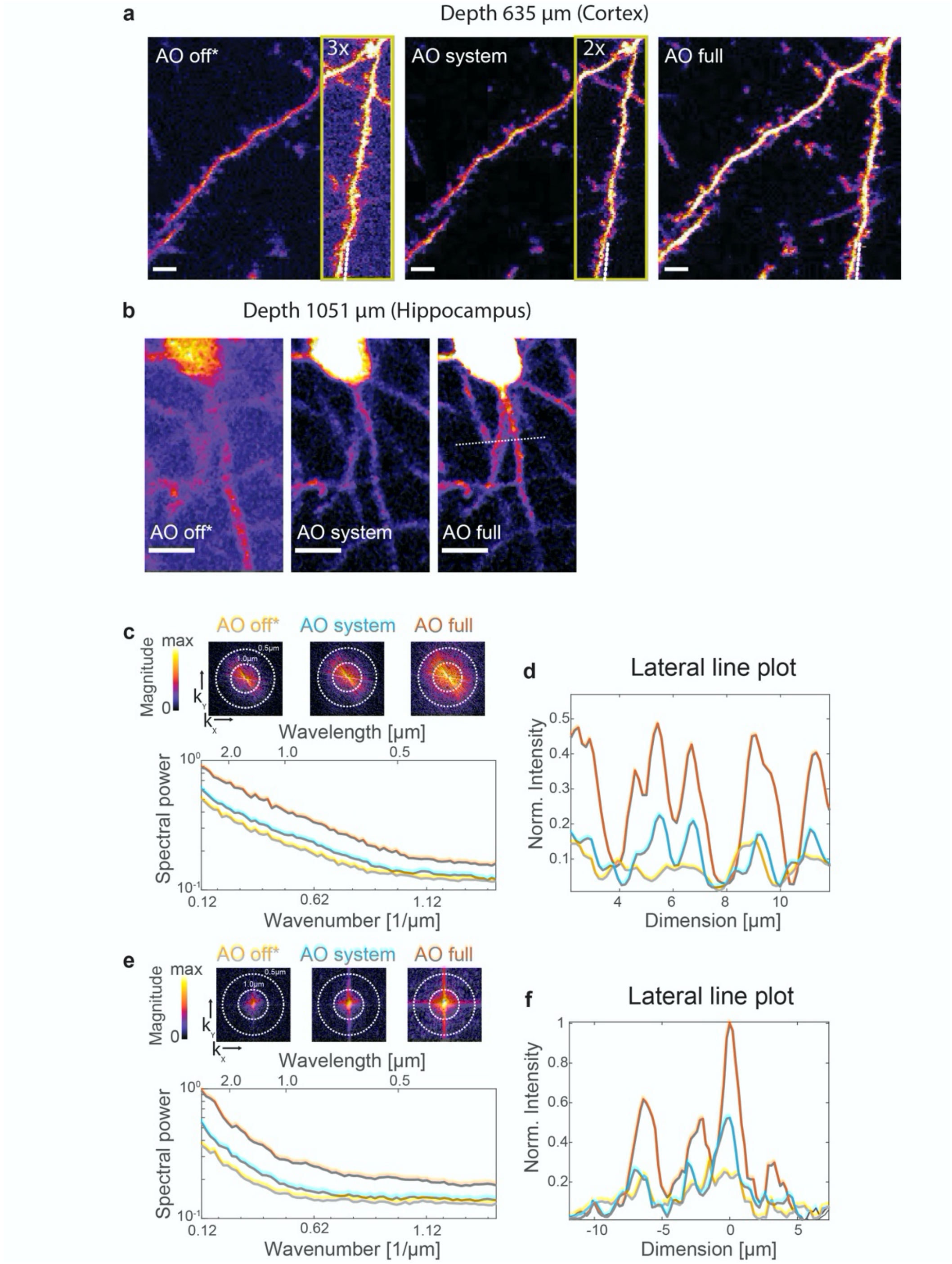

Supplementary Figure 6: Image quality improvement for in-vivo imaging with ECG-gated acquisition and aberration correction. Images were recorded in Thy1-EGFP(M) mice with three different conditions. AO off*: no wavefront correction and no acquisition gating; AO system: wavefront correction of system aberrations and cardiac gated image acquisition; AO full: wavefront correction of system and brain tissue aberrations and cardiac gated image acquisition. Maximum intensity projection images for the three different conditions at **(a)** layer V (scale bar 5µm) and **(b)** in the hippocampus (scale bar 10µm). FOVs indicated by green box were intensity scaled by stated factor to improve visibility. **(c)** and **(e)** (Top) Spectral power map as a function of spatial frequency (wavenumber) and (bottom) average radial profile of spectral power maps for maximum intensity images and **(d)** and **(f)** lateral intensity plot along dotted line indicated in (a) and (b), respectively. Representative results of (n=5) and (n=15) experiments in (a,b), respectively.

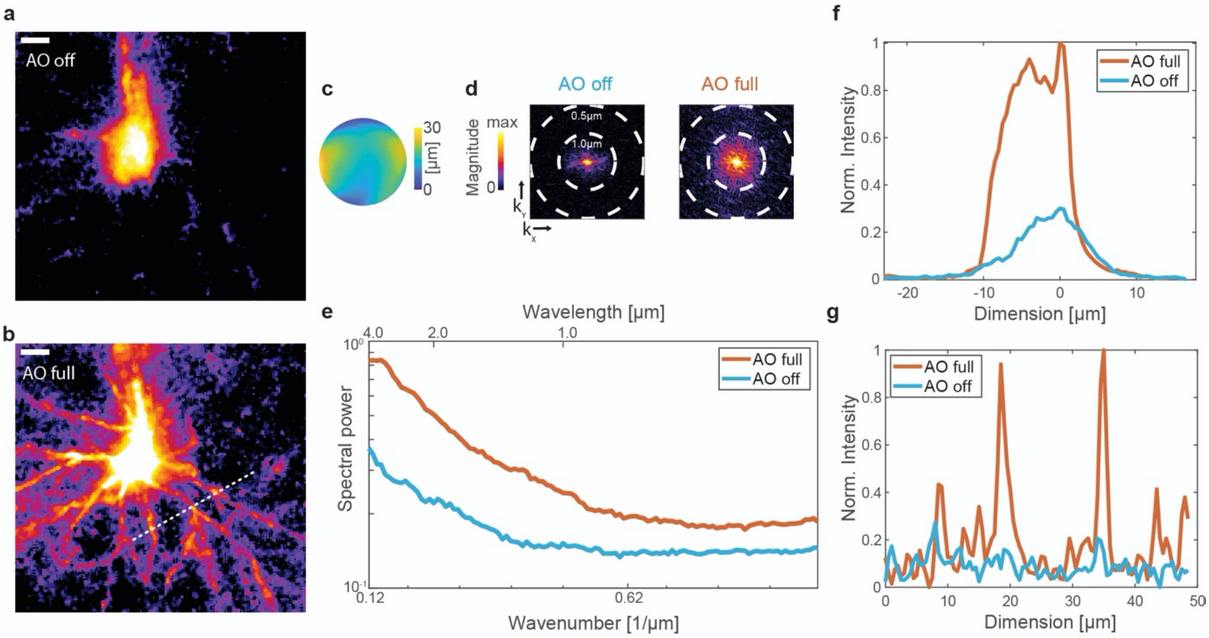

Supplementary Figure 7: Indirect adaptive optics correction and three-photon microscopy imaging of dendrites and somata through a ~40μm thick thinned skull at 400μm depth below the pia. Images were recorded in Thy1-EGFP(M) mice with two different conditions: AO off: no aberration correction and AO full aberration correction of Zernike modes up to the 35th mode excluding the tip/tilt and defocus mode. Maximum intensity projection images **(a)** without and **(b)** with aberration correction. **(c)** Corrected wavefront (WF). **(d)** Spectral power map as a function of spatial frequency (wavenumber) of images in (a) and (b). **(e)** Average radial profile of spectral power maps. **(f)** Lateral (x,y) intensity profile along somata and **(g)** along dendrites indicated in (b) by white dotted line. Scale bar in (a-b) 5μm. Representative result of (n=2) experiments.

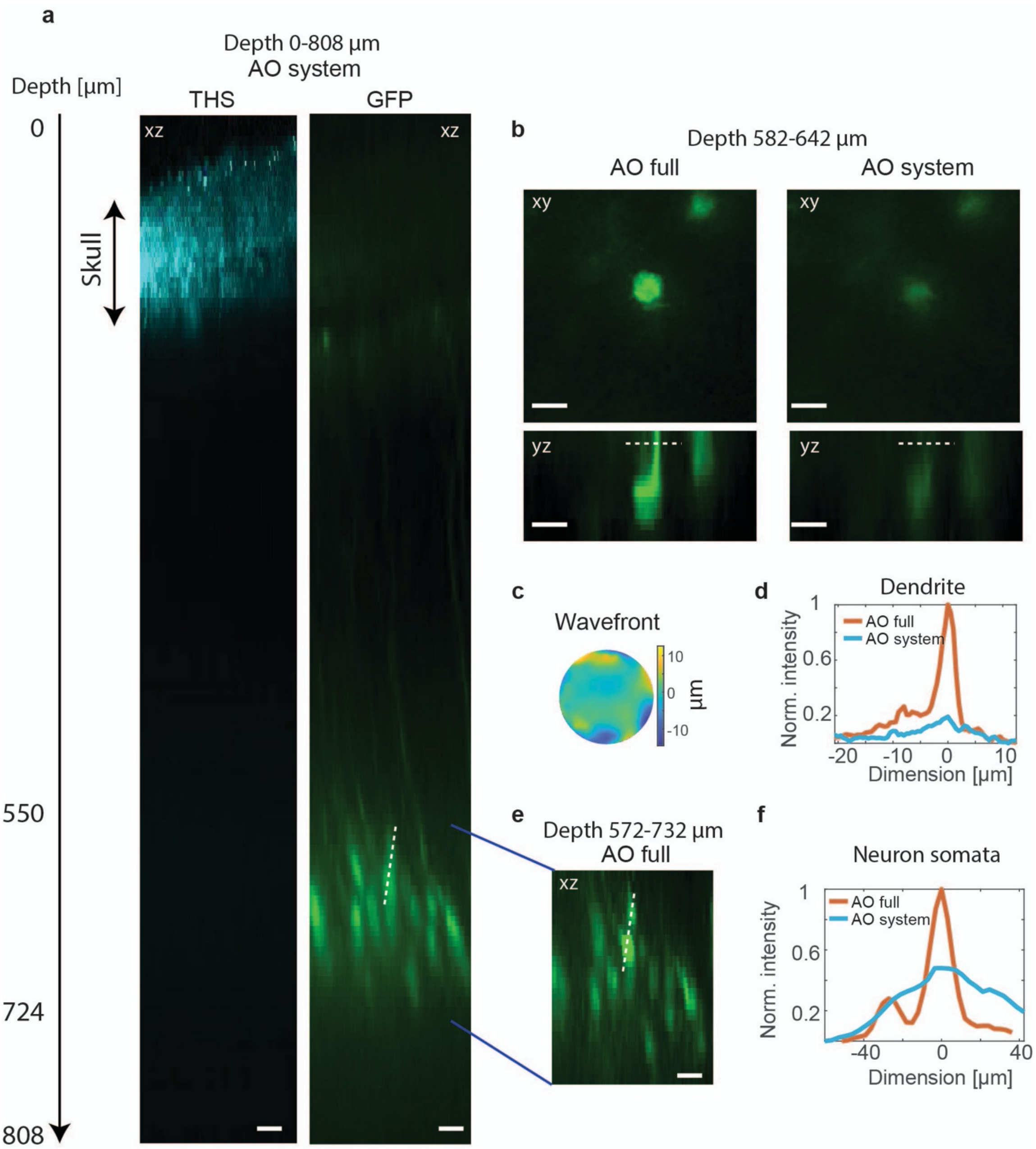

Supplementary Figure 8: Three-photon adaptive optics microscopy at 1300nm excitation through intact skull of ~110 µm thickness of a Thy1-GFP mouse. (a) Orthogonal view of third harmonic signal (cyan) and GFP neuron signal (green) recorded with system aberration correction down to ~800 µm depth below the skull. **(b)** Maximum intensity projection of lateral (top) and orthogonal (bottom) view for system and full aberration correction. **(c)** Correction wavefront displayed on deformable mirror. **(d)** Intensity line pot across dendrite indicate in (b) by white dotted line**. (e)** Orthogonal projection with full aberration correction corresponding to indicated FOV in (a). **(f)** Intensity line plot across somata indicated in (a,e) by white dotted line. Scale bars 20µm. This proof-of-principle experiment was performed on one mouse (n=1).

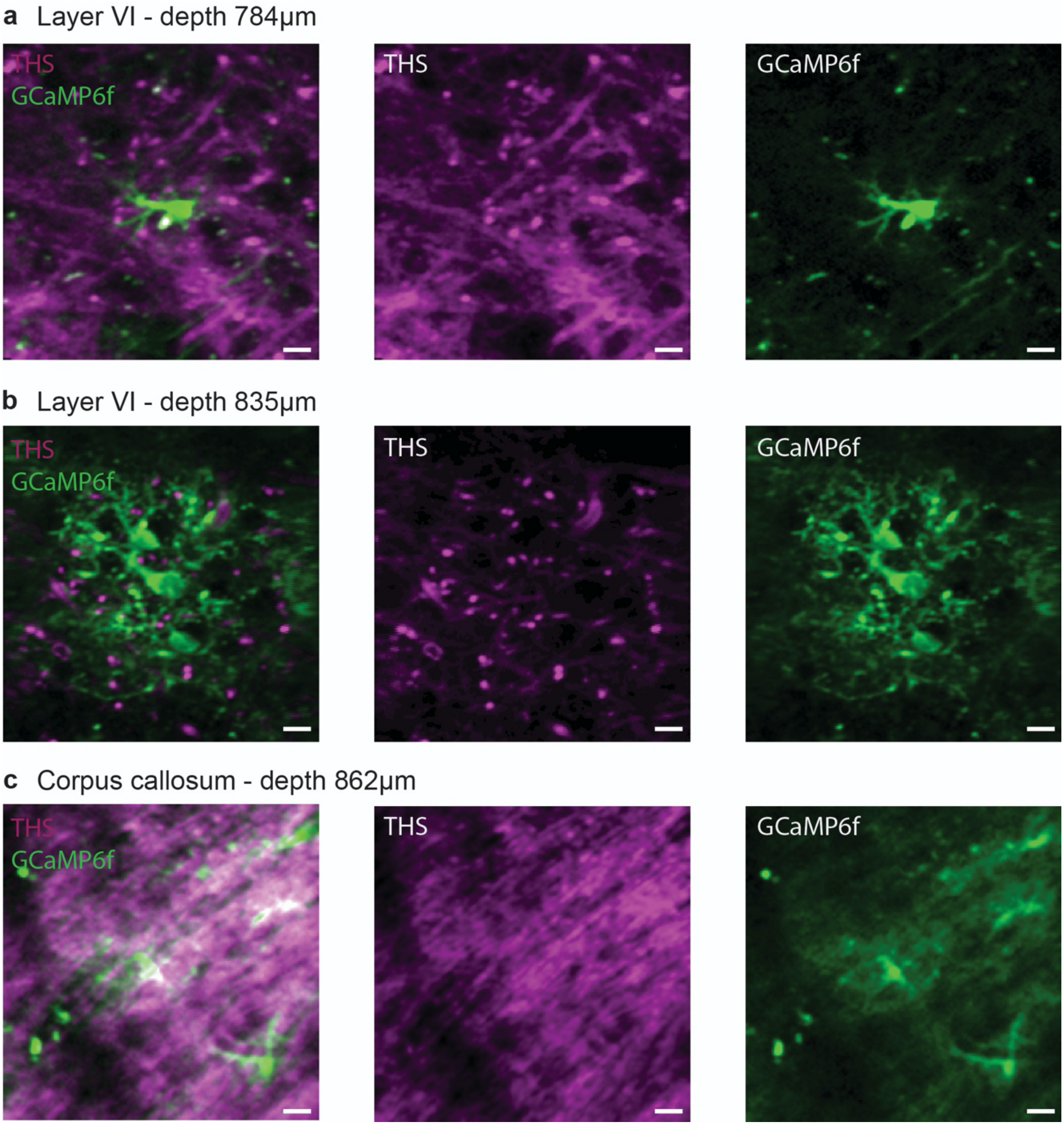

Supplementary Figure 9: GCaMP6f labeled astrocytes and THS in grey and white matter. Intrinsic THS (magenta) generated at the interface of materials with different third-order susceptibility such as blood vessels and the myelinated axons running through the corpus callosum (white matter, c). GCaMP6f astrocytes are displayed in green. (a), (b) and (c) correspond to images in **Fig. 3d,c** and **b**, respectively. Scale bar 10µm. Representative result of (n=5), (n=5) and (n=4) cells in (a-c), respectively.

Supplementary Tables

Supplementary Table 1: Acquisition parameters for high resolution in-vivo imaging at different depth shown in this work.

Fig.	Imaging depth (µm)	Power at sample surface (mW)	Image FOV (µm^3)	Number of pixels / z-depth increment (µm)	Pixel dwell time (µs)	Number of frames averaged	Frame Rate (Hz) / for time series	Number of frames / for time series	Image post-processing for visualization
1b	0-1200	0.5-22	215x215x1200	512x512/4	7.5	3			3x3 median
1c	701	-	50x50	512x512	12.5	1	1.09	15	3x3 median
2a	822	14	184x184x30	512x512/1.5	37.5	5			3x3 median/ Lateral magnification (xy) first scaled by 2, then 3x3 median
2c	964-1076	25-30	126x126x112	256x256/4	20	5			3x3x3 median
2e	1243-1423	45-90	126x126x112	256x256/4	20	5			3x3x3 median
3b	862	24	114x114	256x256	10	1	1.37	300	median intensity projection
3c	835	14	135x135	256x256	7.5	1	1.87	400	median intensity projection
3d	784	9	104x104	256x256	10	1	1.37	500	median intensity projection
**Supp. Fig.**									
1b(left)	539	4	220x220	512x512	15	1	0.23	15	3x3 median
1b(middle)	Same dataset shown in Fig. 1c								3x3 median
1b(right)	1080	22	70x70	128x128	25	1	2.18	50	3x3median
1e	Same dataset shown in Fig. 1c								
1g	1081	22	42x42	256x256	25	1	0.55	10	raw
3	Brain slice	-	130x130x20	512x512/2	-	2			raw
4	653	10	135x135x30	512x512/2	25	3			raw
6a	635	10	30x30x6	206x206/2	25	3			raw
6b	1050	30	31x50x10	143x229/2	17.5	5			raw
7	400	18	125x125x30	256x256/2	10	5			3x3 median
8	0-816	Max 250	126x126x816	256x256x3	20	5			smooth
**Extended Data Fig.**									
3	0-1450	0.5-90	126x126x1450	256x256/4	20	5			3x3x3 median
4 a	877-1193	28-90	106x106x316	256x256x4	20	4			3x3 median
4d	970-1020	60	86x86x50	256x256x2	20	4			3x3x3 median
6	430-830	11-18	360x360 - x	265x265x(3-4)	10	3			3x3x3median
7	923-1196	30-50	360 x 360 -x	265x265x(3-4)	10	3			3x3x3median
9 b	635	2.11-3.5	100x100x32	512x512x2	10	3			smooth
**Supp. Video**									
1	Same dataset shown in Fig. 1b								Imaris
2	Same dataset shown in Fig. 1c								smooth
3	Same dataset shown in Fig. 2a								smooth
4	817	9-11	164x164x54	512x512/2	20	3			smooth
5	Same dataset shown in Fig. 3b								raw
6	Same dataset shown in Fig. 3d								smooth/ AVG 5 frames/ scaled by 3

7	782	26	164x164	256x256	7.5	1	1.37	300	smooth/ AVG 5 frames/ scaled by 3
8	767	28	70x70	256x256	7.5	1	1.37	500	smooth/ AVG 5 frames/ scaled by 2
9	Same dataset shown in Ext. Data Fig. 3								
10	Same dataset shown in Fig. 2 c,e								

Supplementary Table 2: Acquisition parameters for adaptive optics correction at different depth shown in this work.

**Fig.**	**Imaging depth (µm)**	**Imaging power at surface (mW)**	**Frame rate during AO optimization (Hz)**	**Number of modes corrected**	**Iteration**	**Total number of frames**
2a	822	10	38	Z5-Z33	3	551
2c,e	1047	13	15	Z4-Z36	2	320
3d	804	9	20	Z4-Z22	3	361
**Supp. Fig**						
3	Brain slice	-	38	Z5-Z22	5	522
4	653	5	11	Z5-Z22	3	342
6a	635	5	11	Z5-Z22	3	342
6b	1050	20	38	Z4-Z22	2	266
7	400	-	11	Z4-Z36	3	627
8	617	175	15	Z4-Z36	3	480
**Extended Data Fig.**						
4	100					
6/7	429-1196	5 - 20	15	Z4-Z21 Z4-Z36 Z4-Z61	3	255-855
**Supp. Video**		175	15	Z4-Z36	3	480
3	Same dataset shown in Fig. 2a					
4	817					
6	Same dataset shown in Fig. 3d	4	38	Z4-Z36	3	627
7	826					
8	735	20	20	Z4-Z22	3	361
10	Same dataset shown in Fig. 2 c,e	20	20	Z4-Z22	3	361
