# Supplementary material for: High-resolution structural and functional deep brain imaging using adaptive optics three-photon microscopy
Source: Nat Methods. 2021 Sep 30;18(10):1253–8. doi: 10.1038/s41592-021-01257-6 (PMC8490155; doi:10.1038/s41592-021-01257-6)
Supplement: Supplementary file 2 — Reporting Summary [file 41592_2021_1257_MOESM2_ESM.pdf]

nature research

Corresponding author(s): Robert Prevedel

Reporting Summary

Nature Research wishes to improve the reproducibility of the work that we publish. This form provides structure for consistency and transparency in reporting. For further information on Nature Research policies, see Authors & Referees and the Editorial Policy Checklist.

Statistical parameters

When statistical analyses are reported, confirm that the following items are present in the relevant location (e.g. figure legend, table legend, main text, or Methods section).

n/a	Confirmed	
☐	☒	The exact sample size (*n*) for each experimental group/condition, given as a discrete number and unit of measurement
☐	☒	An indication of whether measurements were taken from distinct samples or whether the same sample was measured repeatedly
☐	☒	The statistical test(s) used AND whether they are one- or two-sided<br>*Only common tests should be described solely by name; describe more complex techniques in the Methods section.*
☒	☐	A description of all covariates tested
☒	☐	A description of any assumptions or corrections, such as tests of normality and adjustment for multiple comparisons
☐	☒	A full description of the statistics including central tendency (e.g. means) or other basic estimates (e.g. regression coefficient) AND variation (e.g. standard deviation) or associated estimates of uncertainty (e.g. confidence intervals)
☐	☒	For null hypothesis testing, the test statistic (e.g. *F*, *t*, *r*) with confidence intervals, effect sizes, degrees of freedom and *P* value noted<br>*Give P values as exact values whenever suitable.*
☒	☐	For Bayesian analysis, information on the choice of priors and Markov chain Monte Carlo settings
☒	☐	For hierarchical and complex designs, identification of the appropriate level for tests and full reporting of outcomes
☒	☐	Estimates of effect sizes (e.g. Cohen's *d*, Pearson's *r*), indicating how they were calculated
☐	☒	Clearly defined error bars<br>*State explicitly what error bars represent (e.g. SD, SE, CI)*

Our web collection on statistics for biologists may be useful.

Software and code

Policy information about availability of computer code

Data collection	Custom written Matlab 2016a microscope control software (based on ScanImage from Vidrio Technologies), as well as Vivado (for FPGA programming).
Data analysis	Custom written Matlab 2018a,b scripts, Fiji 1.52i scripts, code is available from the corresponding authors on request.

For manuscripts utilizing custom algorithms or software that are central to the research but not yet described in published literature, software must be made available to editors/reviewers upon request. We strongly encourage code deposition in a community repository (e.g. GitHub). See the Nature Research guidelines for submitting code & software for further information.

Data

Policy information about availability of data

All manuscripts must include a data availability statement. This statement should provide the following information, where applicable:
- Accession codes, unique identifiers, or web links for publicly available datasets
- A list of figures that have associated raw data
- A description of any restrictions on data availability

The main datasets generated and/or analysed during this study are available either as source data files or at 10.5281/zenodo.5060975.

Field-specific reporting

Please select the best fit for your research. If you are not sure, read the appropriate sections before making your selection.

☒ Life sciences ☐ Behavioural & social sciences ☐ Ecological, evolutionary & environmental sciences

For a reference copy of the document with all sections, see nature.com/authors/policies/ReportingSummary-flat.pdf

Life sciences study design

All studies must disclose on these points even when the disclosure is negative.

Sample size	This work focused on the development of a new imaging technique. The performance of the microscope was validated on imaging live neurons and astrocytes and by analyzing the point-spread function of the microscope and the resulting image quality. The sample size was 35 which is the number of total imaging sessions and recordings, and was not based on any sample size calculation. In general, the imaging was repeated between 2 to 11 times each on 16 separate mice, all of which produced comparable data quality. These numbers are indicated in the main text. This sample size was sufficient in our opinion since we were demonstrating a microscope's performance and were not investigating a biological question.
Data exclusions	No data was intentionally excluded from the study. Representative data sets were chosen for the Figures.
Replication	We repeated the in-vivo imaging experiments multiple times (30+) on a total of 16 individual mice (13 Thy-1-GFP, 3 AAV-GFAP-GCaMP6f) and the results were reproducible, i.e. they yielded image datasets of comparable quality.
Randomization	No randomization was applied as this was not essential for our study which concerned the demonstration of a new microscopy technique. We pre-screened mice and their cranial windows for good optical transparency.
Blinding	In principle we were blinded to any group allocation since the mice were chosen randomly or by good window quality and the outcome of our study, i.e. the demonstration of a new microscopy technique, is independent of the biological sample studied.

Reporting for specific materials, systems and methods

Materials & experimental systems

n/a	Involved in the study
☒	☐ Unique biological materials
☒	☐ Antibodies
☒	☐ Eukaryotic cell lines
☒	☐ Palaeontology
☐	☒ Animals and other organisms
☒	☐ Human research participants

Methods

n/a	Involved in the study
☒	☐ ChIP-seq
☒	☐ Flow cytometry
☒	☐ MRI-based neuroimaging

Animals and other organisms

Policy information about studies involving animals; ARRIVE guidelines recommended for reporting animal research

Laboratory animals	This work followed the European Communities Council Directive (2010/63/EU) to minimize animal pain and discomfort. All procedures described in this manuscript were approved by EMBL's committee for animal welfare and institutional animal care and use (IACUC), under protocol number RP170001. Experiments were performed on C57Bl6/j or homozygous Thy1-EGFP-M (Jax No: 007788) transgenic mice from the EMBL Heidelberg core colonies (male and female, 7 to 24 weeks old). During the course of the study mice were housed in groups of 1-5 in makrolon type 2L or 3H cages, in ventilated racks at room temperature and 50% humidity while kept at a 12/12 hr light dark cycle. Food and water was available ad libitum.
Wild animals	The study did not involve wild animals.
Field-collected samples	The study did not involve field-collected samples.
