# Supplementary figures and images for: High-resolution structural and functional deep brain imaging using adaptive optics three-photon microscopy
Source: Nat Methods. 2021 Sep 30;18(10):1253–8. doi: 10.1038/s41592-021-01257-6 (PMC8490155; doi:10.1038/s41592-021-01257-6)

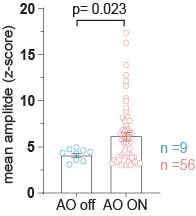

Supplement: Source Data Fig. 3 — Source data for Fig. 3 [file 41592_2021_1257_MOESM15_ESM.zip › source data/Bar graph/barplot.jpg]

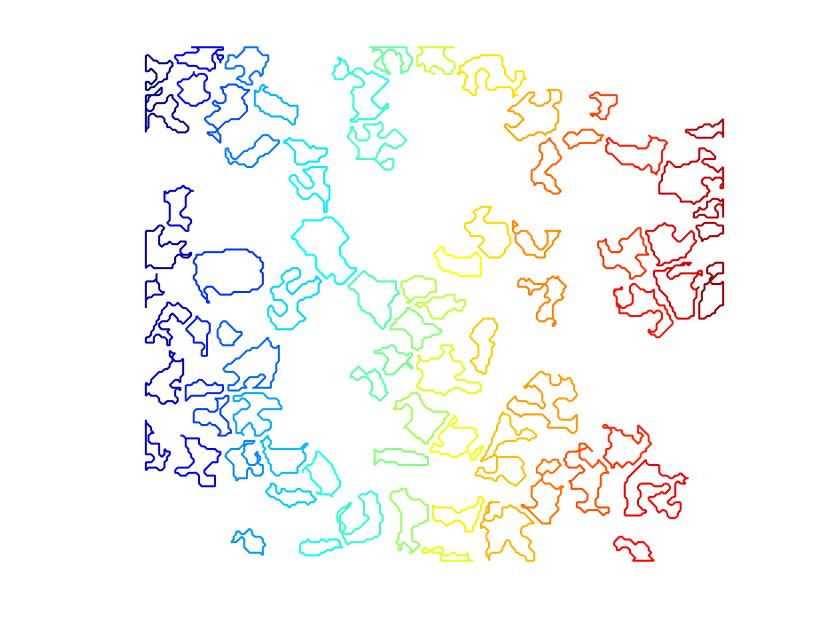

Supplement: Source Data Fig. 3 — Source data for Fig. 3 [file 41592_2021_1257_MOESM15_ESM.zip › source data/Cortex_835um/Domain Map.jpg]

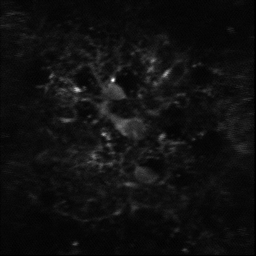

Supplement: Source Data Fig. 3 — Source data for Fig. 3 [file 41592_2021_1257_MOESM15_ESM.zip › source data/Cortex_835um/MED_AVG_Astroyte - 618980 - depth_835um.tif]

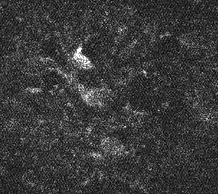

Supplement: Source Data Fig. 3 — Source data for Fig. 3 [file 41592_2021_1257_MOESM15_ESM.zip › source data/Cortex_835um/AVG_Astroyte - 618980 - depth_835um_frrate1.87Hz_bin3_power70_zoom10_00001#2_TregTx1-cropped.tif]

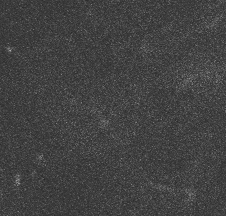

Supplement: Source Data Fig. 3 — Source data for Fig. 3 [file 41592_2021_1257_MOESM15_ESM.zip › source data/CC_862um/AVG_depth_CC_862_power100_bin4_zoom10_frRa1.37_00001.tif #2_TregT-1.tif]

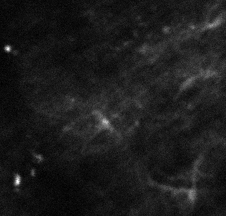

Supplement: Source Data Fig. 3 — Source data for Fig. 3 [file 41592_2021_1257_MOESM15_ESM.zip › source data/CC_862um/MED_AVG_depth_CC_862_power100_bin4_zoom10_frRa1.37]

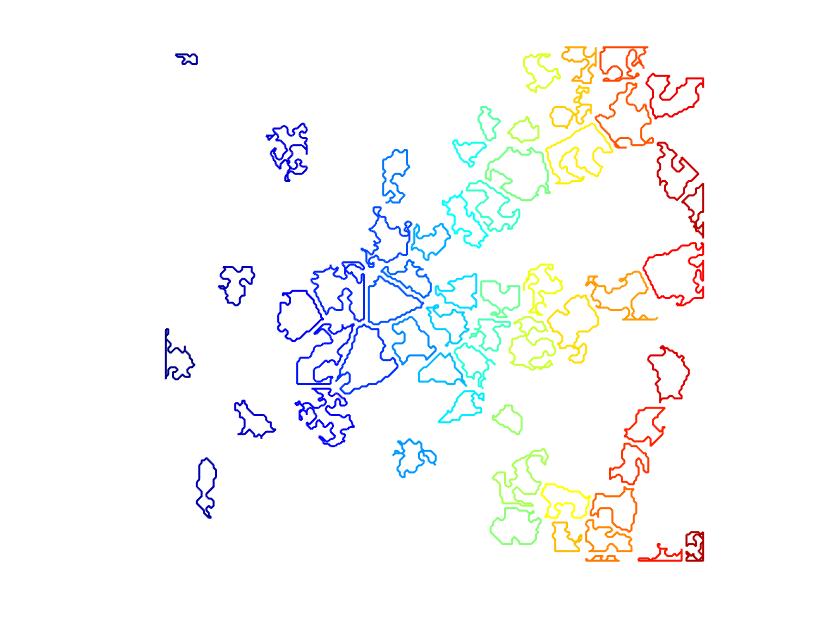

Supplement: Source Data Fig. 3 — Source data for Fig. 3 [file 41592_2021_1257_MOESM15_ESM.zip › source data/CC_862um/Domain Map.jpg]

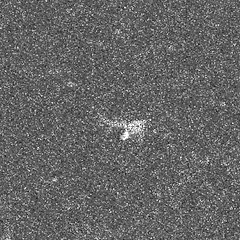

Supplement: Source Data Fig. 3 — Source data for Fig. 3 [file 41592_2021_1257_MOESM15_ESM.zip › source data/Cortex_784um/AVG_Depth784_AO_off_AO_on_279.tif]

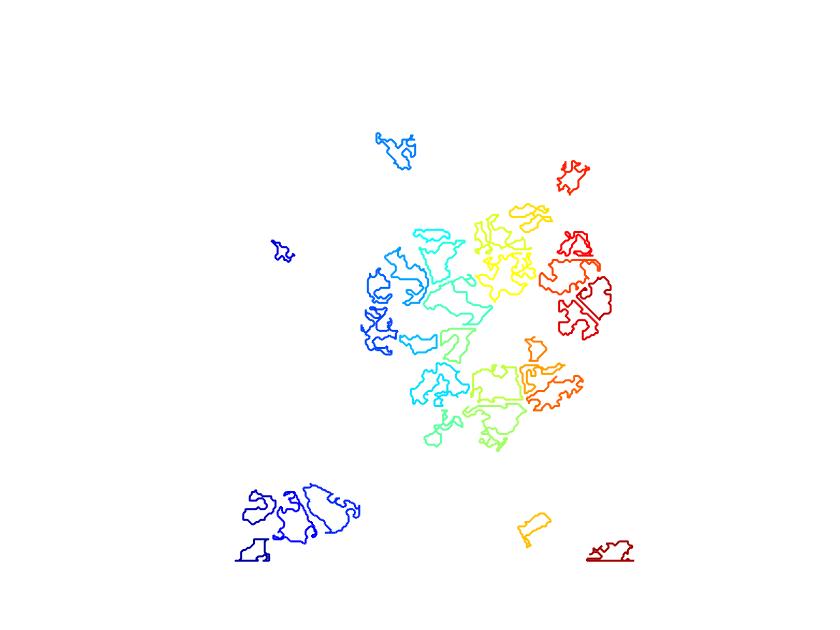

Supplement: Source Data Fig. 3 — Source data for Fig. 3 [file 41592_2021_1257_MOESM15_ESM.zip › source data/Cortex_784um/domain map.jpg]

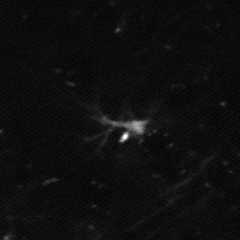

Supplement: Source Data Fig. 3 — Source data for Fig. 3 [file 41592_2021_1257_MOESM15_ESM.zip › source data/Cortex_784um/MED_AVG_Depth784_AO_off_AO_on_279.tif]
